# Compositional Studies and Bioactivity-Guided Fractionation of Acetylcholinesterase Inhibitors in *Papaver nudicaule* from Mongolia—The Role of Amurensinine

**DOI:** 10.3390/molecules31132249

**Published:** 2026-06-26

**Authors:** Enkhtuul Bayarsaikhan, Magdalena Maciejewska-Turska, Maryna Koval, Tomasz Laskowski, Magdalena Lasota, Otgonbaatar Urjin, Davaadagva Damdinjav, Katarzyna Gaweł-Bęben, Wirginia Kukula-Koch, Daariimaa Khurelbat

**Affiliations:** 1 Department of Pharmaceutical Chemistry and Pharmacognosy, School of Pharmacy, Mongolian National University of Medical Sciences, Ard Ayush Str. 48/111, Ulaanbaatar 14210, Mongolia; enkhtuul.b@mnums.edu.mn (E.B.); otgonbaatar0921@gmail.com (O.U.); davaadagva@mnums.edu.mn (D.D.); daariimaa@mnums.edu.mn (D.K.); 2 Department of Pharmacognosy with Medicinal Plants Garden, Medical University of Lublin, 1 Chodzki Str., 20-093 Lublin, Poland; magdalena.maciejewska-turska@umlub.edu.pl; 3 Doctoral School of the Medical University of Lublin, Medical University of Lublin, 7 Chodzki Str., 20-093 Lublin, Poland; maryna.koval@umlub.edu.pl; 4 Department of Pharmaceutical Technology and Biochemistry and BioTechMed Centre, Faculty of Chemistry, Gdańsk University of Technology, Gabriela Narutowicza Str. 11/12, 80-233 Gdańsk, Poland; tomasz.laskowski@pg.edu.pl; 5 Department of Cosmetology, University of Information Technology and Management in Rzeszów, Sucharskiego 2, 35-225 Rzeszów, Poland; mlasota@wsiz.edu.pl (M.L.); kagawel@wsiz.edu.pl (K.G.-B.)

**Keywords:** Mongolian plants, poppy, acetylcholinesterase inhibitors, centrifugal partition chromatography (CPC), isoquinoline alkaloids, amurensinine, NMR, LC-MS

## Abstract

*Papaver nudicaule* L. is a medicinal plant traditionally used in Asian ethnomedicine, yet its phytochemical composition and biological activity remain insufficiently explored. This study bridges phytochemistry and neuroactive potential. It includes metabolite profiling with bioactivity-guided fractionation, performed to evaluate its potential as a source of acetylcholinesterase (AChE) inhibitors. The methanolic extract of the aerial parts was analysed using HPLC–ESI–QTOF-MS/MS, which enabled the tentative identification of 34 compounds, predominantly isoquinoline alkaloids and flavonoid derivatives. The extract was subsequently fractionated by centrifugal partition chromatography (CPC) using an optimised biphasic solvent system, yielding fractions enriched in alkaloid constituents. The obtained fractions were evaluated for AChE inhibitory activity, revealing significantly higher activity than that of the crude extract. The most active fractions exhibited marked inhibition based on the comparison with the reference compound berberine, indicating effective enrichment of bioactive metabolites. Further analysis demonstrated that the activity of the most potent fraction was associated with the presence of amurensinine, which was purified by preparative HPLC and subsequently identified by NMR and LC-MS. The *Papaver nudicaule* extract showed no significant cytotoxicity toward SH-SY5Y neuronal cells up to 200 µg/mL, whereas the amurensinine-containing fraction reduced cell viability only at higher concentrations (≥100 µg/mL). Notably, when expressed in the micromolar range, this effect corresponds to relatively weak cytotoxicity, suggesting a potential safety margin at lower, biologically relevant concentrations. These findings demonstrate that *P. nudicaule* possesses a highly diverse alkaloid profile and represents a promising natural source of compounds with potential relevance for the development of agents targeting neurodegenerative disorders.

## 1. Introduction

The Papaveraceae family comprises approximately 44 genera and nearly 760 species of flowering plants distributed worldwide [1]. Among them, the genus Papaver, first described by Carl Linnaeus in Species Plantarum in 1753, is one of the most chemically and pharmacologically significant groups within this botanical family [2]. Species in this genus are recognised as valuable sources of biologically active secondary metabolites, particularly alkaloids with diverse pharmacological properties.

*Papaver nudicaule* L., commonly known as the Iceland poppy, is a perennial plant widely distributed in Central and Eastern Siberia, the Far East, Korea, China, and Central Asia [1]. The species occurs in diverse habitats, including meadows, rocky areas, feather grass steppes, sandy riverbanks, gravelly soils, and shrublands [1]. In traditional Mongolian and Tibetan medicine, preparations derived from *P. nudicaule* have long been used to treat various disorders, including headaches, dysmenorrhea, dysentery, gastrointestinal diseases, fever, and wound healing. Furthermore, the plant is included in several traditional medicinal formulations used to treat inflammatory conditions, vascular injuries, and digestive system disorders [3,4,5,6]. These ethnopharmacological applications suggest the presence of biologically active metabolites responsible for the plant’s therapeutic effects.

Phytochemical studies on *Papaver* species have revealed a wide range of secondary metabolites, among which alkaloids are among the most characteristic and pharmacologically important groups. In particular, isoquinoline alkaloids are widely distributed within the Papaveraceae family and are responsible for many of the biological activities attributed to these plants. Previous studies on *P. nudicaule* have identified numerous isoquinoline derivatives, including (-)-8,14-dihydropalmatine, amurensine, (-)-amurensinine, (-)-dihydroamuramine, (-)-O-methylthalizopavine, (-)-palmatine, (+)-amuramine, 8,14-dihydroamuramine, allocryptopine, amurensinine-α-N-oxide, amurensinine-β-N-oxide, and pseudoprotopine [7]. Additionally, phytochemical investigations have led to the isolation of novel compounds, such as the promorphinane alkaloid (-)-8,14-dihydroflavinantine from the aerial parts of the plant, together with several known isoquinoline alkaloids including (+)-amuronine, pseudoprotopine, allocryptopine, (-)-dihydroamuronine, (-)-amurensinine N-oxide A, and (-)-amurensinine N-oxide B [8]. These findings demonstrate the remarkable chemical diversity of *P. nudicaule* and highlight its potential as a source of pharmacologically relevant natural compounds.

In addition to alkaloids, other classes of secondary metabolites have also been identified in *P. nudicaule*. Studies investigating differently colored cultivars of this species have revealed the presence of flavonoids, carotenoids, and unique pigment compounds known as nudicaulins. Chromatographic and spectroscopic analyses confirmed the presence of gossypetin glycosides and kaempferol glycosides as major metabolites in different plant organs, including petals, stamens, and capsules [9]. Earlier research also reported the occurrence of gossypitrin and several kaempferol derivatives in the yellow petals of Iceland poppy [10]. Furthermore, nudicaulins I–VIII were identified and quantified in yellow and orange flowers of *P. nudicaule* and in related species, such as *Papaver alpinum* [11]. More recent investigations combining transcriptomic, proteomic, and metabolomic approaches have provided insights into the biosynthetic pathways responsible for nudicaulin formation in the petals of *P. nudicaule* [12]. Despite these studies, the comprehensive characterisation of the metabolite profile of this species remains limited, and the biological activities of many individual compounds isolated from *P. nudicaule* have not yet been sufficiently investigated.

Isoquinoline alkaloids have attracted considerable scientific interest due to their diverse pharmacological properties, including antimicrobial, anti-inflammatory, antioxidant, and neuroprotective activities. Of particular importance is their potential application in treating neurodegenerative diseases [13]. Several representatives of this group, especially isopavine alkaloids characterised by a tetracyclic tetrahydroisoquinoline core structure, have been reported to exhibit pharmacological activity relevant to neurological disorders such as Alzheimer’s disease (AD), Parkinson’s disease, Huntington’s chorea, amyotrophic lateral sclerosis, and Down syndrome. In addition, both natural and synthetic analogues of these compounds have demonstrated significant affinity for opioid receptors [14].

One of the key therapeutic targets in AD is acetylcholinesterase (AChE), the enzyme responsible for the hydrolysis of acetylcholine in the synaptic cleft. The inhibition of AChE leads to increased acetylcholine levels and improved cholinergic neurotransmission, which may alleviate cognitive symptoms associated with neurodegenerative disorders [15]. Consequently, the search for natural AChE inhibitors has become an important area of research in medicinal chemistry and pharmacognosy, and numerous plant-derived alkaloids have been investigated as potential therapeutic agents [16,17].

The isolation of individual alkaloids from complex plant matrices remains a major challenge in natural product chemistry. Conventional chromatographic techniques typically employ solid stationary phases, which may result in irreversible adsorption of alkaloids, peak tailing, and reduced recovery of target compounds. In recent years, liquid–liquid chromatographic techniques have gained increasing attention as alternative methods for natural product isolation. Among them, centrifugal partition chromatography (CPC) represents an advanced countercurrent separation technique that operates without a solid stationary phase. The absence of a solid adsorbent minimises analyte loss and improves recovery efficiency, making CPC particularly suitable for the isolation of alkaloids from plant extracts. Moreover, CPC enables efficient and scalable separations and has been successfully applied for the isolation of isoquinoline alkaloids from various plant matrices [18,19].

Despite the reported presence of numerous secondary metabolites in *P. nudicaule*, comprehensive metabolite profiling combined with preparative isolation of individual compounds remains scarce. Furthermore, the biological activities of isolated metabolites from this species, particularly their potential effects on enzymes relevant to neurodegenerative diseases, have not yet been systematically explored.

Therefore, the present study aimed to investigate the phytochemical profile of *P. nudicaule* and to fractionate its extract using centrifugal partition chromatography, an efficient method for the isolation of alkaloids. Subsequently, the obtained fractions and isolated metabolites were evaluated for their inhibitory activity against AChE to assess their potential relevance to the development of natural compounds that support the therapy of AD. In these studies berberine was selected as a reference inhibitor due to its well-documented acetylcholinesterase inhibitory activity and its structural relevance to the isoquinoline alkaloids identified in the studied extract. As a representative of protoberberine-type alkaloids commonly occurring in *Papaver* species, it provided a suitable benchmark for evaluating the activity of related natural compounds.

## 2. Results and Discussion

The phytochemical investigation of *P. nudicaule* was performed using a combination of chromatographic and spectrometric techniques. First, HPLC–ESI–QTOF-MS/MS analysis was applied to characterise the metabolite profile of the extract and identify its major constituents. Based on the results obtained, centrifugal partition chromatography (CPC) was subsequently used to fractionate the extract, and the resulting fractions were evaluated for AChE inhibitory activity.

### 2.1. Compositional Studies of P. Nudicaule Extract by HPLC-ESI-QTOF-MS/MS

**Figure 1:**
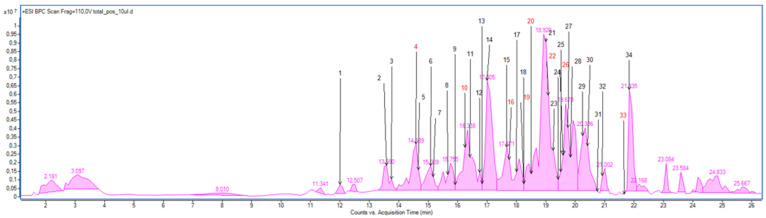
The mass chromatogram of *P. nudicaule* methanolic extract recorded in the positive (black) and negative (red) ion mode (black and red numbers indicate the *m*/*z* signals obtained at this part of the mass chromatogram that are relevant to those from Table 1; pink values at each peak stand for the retention time).

The qualitative chemical composition of the extract obtained from the aerial parts of *P. nudicaule* was investigated using HPLC–ESI–QTOF-MS/MS analysis based on the recorded mass chromatograms showing a high number of signals and standing for the richness of this plant preparation (see Figure 1).

The compounds’ assignments were based on accurate mass measurements, characteristic fragmentation patterns, and comparisons with previously reported data for compounds occurring in species of the Papaveraceae family. In total, 34 compounds were tentatively identified in the analysed extract (Table 1), including alkaloids and flavonoid derivatives. The chromatographic and mass spectrometric data of the detected metabolites are presented in Table 1, and their MS/MS spectra are shown in the Supplementary File (Table S1). The identified compounds were annotated according to the Metabolomics Standards Initiative (MSI) guidelines. All compounds except from amurensinine were classified as level 2 (putatively annotated compounds) based on accurate mass measurements and MS/MS fragmentation patterns compared with literature and database data. Amurensinine was identified based on the NMR spectrum that was recorded after its purification from the extract.

The investigated plant species is widely distributed in Mongolia, where it has long been used in traditional medicine to treat various ailments under the names “Khurgan zasaa,” “Sharhnii shar,” and “Shar jamen”. The plant is described as having a bitter taste and a cold nature, with antimicrobial, antipyretic, detoxifying, and anti-pneumonic properties. Traditionally, it has been used to treat inflammatory conditions, diarrhoea, dysmenorrhea, acute and chronic gastric inflammation, gastric ulcers, purulent wounds, vascular disorders, oedema, and tendon injuries. *P. nudicaule* is included in traditional formulations such as Govo Jad-5 and Namuu-4 and is sometimes used as a single medicinal agent [3,5]. Given its widespread traditional use, phytochemical investigations and standardisation studies are essential to support its further application as a medicinal plant.

The isoquinoline structure is a well-known key biosynthetic precursor to many bioactive alkaloids from plants [35]. The LC–MS/MS analysis revealed that, benzyltetrahydroisoquinolines, containing a saturated B-ring at the C1–C2 and C3–C4 positions, constitute the dominant group of metabolites present in the in *P. nudicaule* extract. Among this subclass, norcoclaurine (**2**), higenamine-*O*-glucoside (**1**), coclaurine/isococlaurine (**8**), N-methylisococlaurine/N-methylcoclaurine (**9**), reticuline (**13**), 2-methylmagnocurarine (**11**) and magnocurarine (**6**) were identified. These compounds represent early intermediates in the biosynthetic pathway of many isoquinoline alkaloids widely distributed in plants of the family Papaveraceae [36]. Their identification in the extract confirms the typical chemotaxonomic profile of the genus *Papaver*. The LC-MS analysis provided evidence that these compounds share a distinctive fragmentation pattern, characterised by diagnostic fragment ions originating from the neutral loss of small molecules, such as NH(CH_3_)_2_ (−45 Da), NH_2_CH_3_ (−31 Da) and NH_3_ (−17 Da), from the precursor ion with subsequent B-ring opening and cleavage of the isoquinoline skeleton [25,28].

Selecting coclaurine/isococlaurine (**8**) as an example, neutral loss of NH_3_ (−17 Da) from the precursor [M + H]^+^ ion at *m*/*z* 286.1438 (C_17_H_19_NO_3_) formed a product ion at *m*/*z* 269.1168, providing structural information on substituents attached to the nitrogen atom. The presence of an intensive fragment ion at *m*/*z* 107.0490 (C_7_H_7_O; −1.33 ppm), resulting from β-cleavage, indicated the presence of a single hydroxyl substituent in the C-ring [26]. The product ion at *m*/*z* 175.0746 generated via α-cleavage was also observed in the MS/MS spectra of this compound, suggesting the substitution with one hydroxyl and one methoxyl group in the A-ring, consistent with the fragmentation behaviour of magnocurarin (**6**) described by Zuo et al. [25]. For compound 9, four possible structural isomers were considered. The intense fragmentation ion at *m*/*z* 269.1168, formed through the loss of NH_2_CH_3_ from the [M + H]^+^ ion at *m*/*z* 300.1596, indicated N-methylcoclaurine (or N- methylisococlaurine) structure rather than norarmepavine [26]. Moreover, the presence of an intensive product ion at *m*/*z* 107.0489 excluded 6-demethyl-4′-*O*-methyl-N-methylcoclaurine [24].

**Figure 2:**
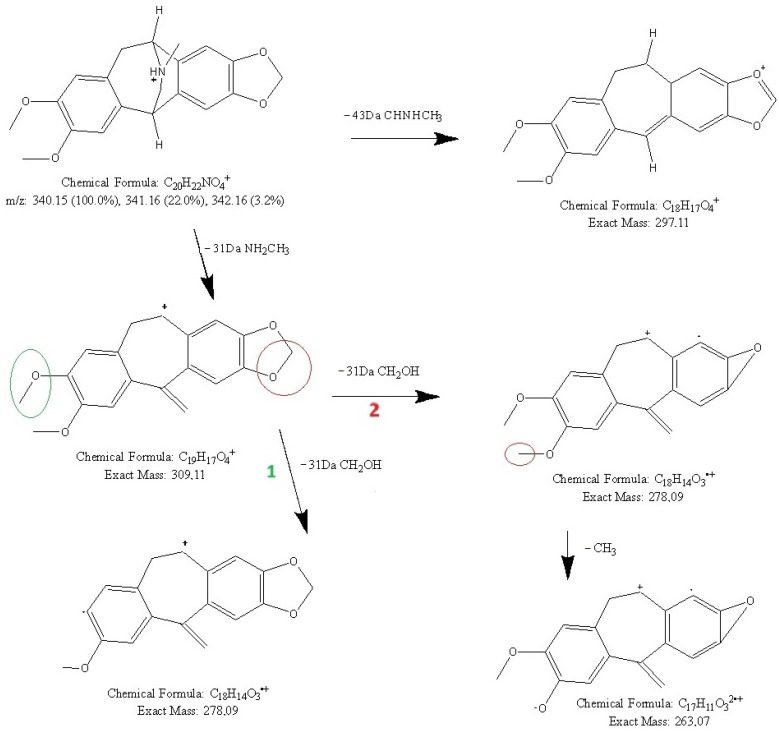
Hypothetical fragmentation pattern proposed to confirm the molecular structure of the isolated compound (amurensinine) (*m*/*z* 340) based on the obtained MS/MS data.

The second most abundant group of alkaloids, biogenetically derived from benzylisoquinolines, is the isopavine alkaloids, which have been identified in several Ranunculaceae and Papaveraceae species [23]. To the best of our knowledge, the fragmentation pathways of these compounds remain scarce in the literature. The extensive spectrometric data obtained from LC–MS analysis and similarity in their fragmentation, under the influence of the CID energy, allowed tentative structural elucidation of eight isopavine alkaloids, namely thalidicine/thalidine (**5**), thalisopavine (**12**), amurensine isomer 1 (**15**) and isomer 2 (**17**), methylthalisopavine (**18**), amurensinine (**21**), amuresinine N-oxide (**23**), reframidine (**28**) and one promorphinane alkaloid—8,14-dihydroflavinantine (**3**) [22,23]. With regard to the mass spectrometric analysis of isolated compound (**21**), the observed precursor ion at *m*/*z* 340.1535 was consistent with the molecular formula of C_20_H_21_NO_4_. A typical neutral loss of NH_2_CH_3_ (−31 Da) from [M + H]^+^ ion with subsequent formation of abundant product ion at *m*/*z* 309.1122 (C_19_H_17_O_4_^+^) was observed in its MS spectrum. Based on the literature data [29,30], it was concluded that, similarly to morphinans, compound 21 underwent a characteristic detachment of the NH_2_CH_3_ amine moiety, causing a decrease in *m*/*z* values by −31 Da. Also, the detachment of an aminomethyl fragment, the CHNH-R, was noted, which is characteristic of all detected isopavine alkaloids. Further fragmentation may occur in two possible ways, involving the subsequent loss of CH_2_OH (−31 Da). The proposed theoretical fragmentation pathway for amurensinine (**21**) is shown in Figure 2.

The aforementioned compound, amurensinine, drew particular attention in this study because it was isolated in high purity during the applied separation process and was shown to exhibit AChE inhibitory activity.

To date, relatively few studies have investigated these compounds in *Papaver* species. Most of the available data originates from the 1990s, when isolation procedures were time-consuming, involved multiple purification steps, and used various hazardous reagents [8].

Another group of detected compounds consisted of protopine-type alkaloids, such as muramine (**24**), dihydrocryptopine/demethylated muramine (**25**), α-allocryptopine (**27**), and cryptopine (**29**), which are typical representatives of the protopine subgroup commonly reported in Papaveraceae species [17]. Similarly, allocryptopine and related derivatives have been reported in a recent molecular network-based study of *P. nudicaule*, confirming the presence of this structural class in the plant material studied [34]. The fragmentation pattern of protopine-type alkaloids was characterised by the loss of small neutral fragments, primarily water and the formation of diagnostic *retro*-Diels–Alder (RDA) ions at *m*/*z* ~190 and ~165, which correspond to C-ring opening [32].

The LC-MS analysis results in some representatives of the protoberberine-type alkaloids. These widely known bioactive constituents are frequently detected in the Papaveraceae family [7]. In the investigated extract, N-methylcanadine (**30**), alborine (**31**) and berberine (**32**) were assigned. Lack of saturation in the C-ring prevents RDA cleavage. Therefore, the MS/MS fragmentation of protoberberine alkaloids, such as berberine, typically involves sequential losses of methyl radical and methoxy groups, producing characteristic ions at *m*/*z* 320, 306, 304, and 292, which are commonly used for the identification of this compound in LC–MS studies.

Furthermore, in addition to the alkaloids, several flavonoid derivatives were identified in the analysed extract in negative ionisation mode. According to previously published studies on *P. nudicaule*, the presence of flavonols, such as kaempferol 3-*O*-β-sophoroside (**18**) and kaempferol 3-*O*-β-sophoroside-7-*O*-β-glucoside (**4**), was confirmed [10].

The MS/MS spectra of these compounds showed typical fragmentation pathways involving C-O bond cleavage and sequential loss of sugar moieties from [M-H]^−^ ions, resulting in the free aglycone. For tetrahydroxyflavones (C_15_H_10_O_6_), characteristic fragment ions at *m*/*z* 285 and 284 were observed, along with informative RDA fragments at *m*/*z* 151 and 133, indicating the presence of kaempferol and luteolin structure, respectively. Two compounds, 22 and 26, that shared a similar fragmentation pattern were assigned to be quercetin-*O*-glucoside isomers, due to the diagnostic ions at *m/z* 301.0342 (C_15_H_10_O_7_) and an intensive fragmentation ion at 151.0027 (C_7_H_4_O_4_), suggesting pentahydroxyflavone core [37].

One low-intensity peak at *m*/*z* 179.0343, corresponding to caffeic acid (**10**), was also observed.

The presence of these metabolites is consistent with previous phytochemical investigations of *P. nudicaule* [16]. The HPLC-MS/MS studies indicate that *P. nudicaule* is a source of various isoquinoline alkaloids from different subgroups, as well as flavonoid glycosides. Earlier studies reported the occurrence of various isoquinolines, including protopine derivatives, allocryptopine, papaverrubines, muramine, and berberine, as well as flavonoids such as gossypetin glucoside and kaempferol derivatives [38]. The predominance of isoquinolines and isopavine alkaloids observed in the present study reflects the characteristic chemotaxonomic features of the Papaveraceae family and confirms that this group of compounds represents the main class of specialized metabolites in *P. nudicaule*. Isoquinolines provide a chemical basis for the traditional medicinal applications of this plant, as many structures are known to exhibit antimicrobial, anti-inflammatory, antioxidant, analgesic, and neuroactive properties [39,40,41,42]. Within this group, isopavine alkaloids remain relatively understudied, with potential anti-neurodegenerative properties. Therefore, efforts to isolate naturally occurring constituents from plant material and to design their synthetic analogues are currently being considered [43].

### 2.2. CPC Fractionation of the Methanolic Extract from P. nudicaule

The metabolite profiling performed by HPLC–ESI–QTOF-MS/MS revealed that the extract of *P. nudicaule* is dominated by isoquinoline alkaloids representing several structural subclasses that are structurally related. This fact necessitates an efficient fractionation technique capable of separating complex mixtures of basic nitrogen-containing compounds.

Therefore, centrifugal partition chromatography (CPC), a liquid–liquid chromatographic technique operating without a solid stationary phase, was selected for extract fractionation. The absence of a solid adsorbent reduces analyte loss and minimises peak tailing, which is particularly advantageous for the isolation of alkaloids. Based on the metabolite profile obtained by HPLC–MS/MS, CPC fractionation was performed to isolate fractions enriched in alkaloid constituents.

#### 2.2.1. The Selection of Conditions for the Fractionation

The partition coefficient values obtained for the tested biphasic solvent systems are presented in Table S2 of the Supplementary File. Among the investigated systems, the mixture composed of n-hexane/n-BuOH/EtOH/H_2_O (3:12:6:15, *v*/*v*/*v*/*v*) exhibited the most favourable distribution of compounds between the phases. In this system, the calculated K_D_ values showed a broad range (approximately 0.18–18.0), indicating significant differences in compound partitioning between the upper and lower phases. Such variability in K_D_ values is advantageous for countercurrent separation, as it suggests the possibility of efficient fractionation of structurally diverse metabolites.

The remaining solvent systems showed considerably narrower K_D_ ranges (e.g., 0.22–0.6) and predominantly low coefficient values, indicating limited partitioning of analytes between the phases and therefore lower separation selectivity. The balanced polarity of the selected solvent system enabled efficient partitioning of alkaloids with intermediate polarity; therefore, it was chosen for further CPC fractionation of the extract.

Still, in counter-current chromatography, optimal separation may be achieved when partition coefficients are within the range of 0.5–1, according to Ito [44]. This range provides a balanced distribution of analytes between the stationary and mobile phases.

#### 2.2.2. The Fractionation of the Extract

**Figure 3:**
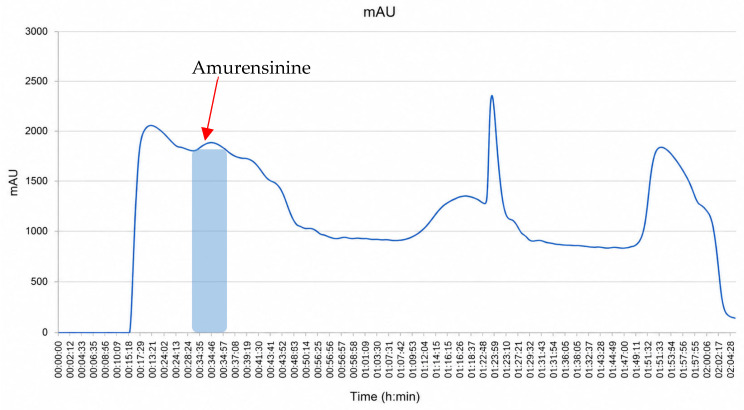
CPC chromatogram of the methanolic extract from *Papaver nudicaule* overground parts.

The CPC separation of the *P. nudicaule* extract was completed within 120 min. The first 60 min were conducted in elution mode, followed by 60 min of extrusion, allowing recovery of compounds remaining in the stationary phase (Figure 3). The applied conditions—including a biphasic solvent system composed of n-hexane–n-butanol–ethanol–water (3:12:6:15, *v*/*v*/*v*/*v*), a rotation speed of 1200 rpm and a mobile phase flow rate of 4 mL/min—provided stable hydrodynamic equilibrium and reproducible separation.

Under these conditions, a selective and well-resolved CPC chromatogram was obtained, enabling efficient fractionation of the crude extract into 30 fractions. Importantly, the separation was achieved without the addition of acidic or basic modifiers such as hydrochloric acid or ammonia. Consequently, the alkaloids remained in their native salt forms, as in the original extract, and no additional neutralisation or evaporation steps were required during fractionation.

Centrifugal partition chromatography (CPC) has emerged as an efficient alternative to conventional column chromatographic techniques for the purification of plant alkaloids. In contrast to classical systems employing solid stationary phases, CPC operates exclusively in a liquid–liquid mode, eliminating irreversible adsorption of analytes and significantly reducing the peak tailing frequently observed during the purification of basic nitrogen-containing compounds. This feature is particularly advantageous for alkaloids, whose interactions with solid supports such as silica gel may lead to partial retention, loss of analytes and decreased recovery. By relying on partitioning between two immiscible liquid phases, CPC enables more reproducible separations and often allows nearly quantitative recovery of target compounds while minimising degradation or structural modification during the purification process [45].

The suitability of CPC for the isolation of alkaloids from complex plant matrices has been demonstrated in numerous studies. For example, Maurya and Srivastava successfully separated the clavine alkaloids lysergol and chanoclavine from *Ipomoea muricata* seeds using a methyl tert-butyl ether–acetonitrile–water solvent system [46]. Similarly, Chollet et al. [47] reported the fractionation of several indole alkaloids from *Catharanthus roseus*, including vindoline and catharanthine, which are used as starting materials for anticancer drugs, thereby confirming that CPC enables efficient purification even with relatively large sample loads. The technique has also been successfully used to isolate isoquinoline alkaloids such as palmatine, jatrorrhizine, columbamine, and pseudocolumbamine from *Enantia chlorantha*, with purities exceeding 95% achieved after CPC separation [48]. Additional reports describe the purification of alkaloids from other plant sources, including *Coptis chinensis* and *Corydalis decumbens*, further demonstrating the versatility of this technique for structurally diverse alkaloid classes [49,50].

Considering these advantages, CPC appears particularly suitable for the fractionation of alkaloid-rich plant extracts such as those obtained from *Papaver* species. Members of the genus *Papaver* are known to contain numerous structurally related isoquinoline alkaloids, which often co-occur in complex mixtures and may exhibit similar chromatographic behaviour in conventional column systems. In this context, the application of CPC enables selective fractionation of such extracts while avoiding adsorption-related losses typical for solid-phase techniques [51].

The conditions used in the present study enabled efficient fractionation of the *P. nudicaule* extract and the generation of well-defined fractions enriched in specific alkaloids, confirming that CPC is a suitable preparative approach for isolating these bioactive compounds from complex plant matrices.

Based on the compositional similarity of the fractions, they were grouped into eight pooled fractions, evaporated to dryness using a rotary evaporator (Eppendorf Concentrator Plus), and subjected to an AChE inhibition assay.

### 2.3. Acetylcholinesterase Inhibitory Activity

**Figure 4:**
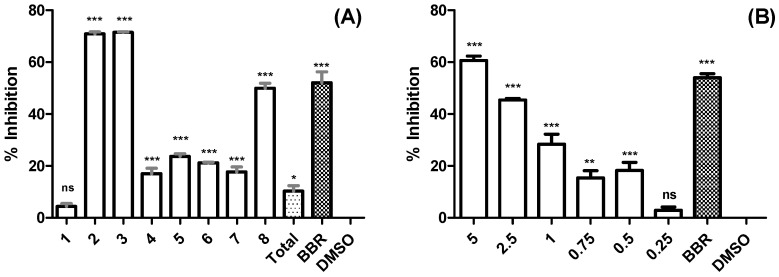
The results of the acetylcholinesterase inhibition assay performed on the CPC fractions, the total extract and the most active fraction ((**A**)—The inhibition of AChE by the CPC fractions (5 mg/mL), total extract (5 mg/mL) and berberine (1 mg/mL); (**B**)—Inhibition of AChE by amurensinine (mg/mL). Error bars represent standard deviations (SD) calculated from three replicate measurements (*n* = 3); * *p* < 0.05; ** *p* < 0.01; *** *p* < 0.001, ns—not significant.

Consequently, each of the collected CPC fractions was further evaluated for inhibitory activity against AChE in vitro to assess its potential relevance to the development of natural compounds that support the therapy of AD. The AChE inhibition assay was performed to evaluate the biological activity of the CPC fractions obtained from the *P. nudicaule* extract. The results revealed clear differences in inhibitory potential among the tested fractions (see Figure 4 below).

It should be noted that different concentrations were used for the tested samples. The CPC fractions, representing complex mixtures of compounds, were evaluated at 5 mg/mL, which is a standard concentration range for extract-based screening to ensure measurable biological effects. In contrast, the reference compound berberine, due to its well-documented potency, was tested at a lower concentration (1 mg/mL).

Therefore, the results presented in Figure 4A should be interpreted in terms of relative activity within each tested group rather than as a direct potency comparison between the fractions and the pure standard.

The most active one (fraction 3) was subsequently purified by preparative HPLC (as described in Section 2.4) and later analysed across different dilutions, as shown in Figure 4B. The mass chromatograms representing the composition of the two most active fractions—fraction 2 and 3 are presented in the Supplementary File (Figures S1 and S2).

The strongest inhibition of AChE was observed for fractions 2 and 3, which exhibited 70.90 ± 1.35% and 71.49 ± 0.32% inhibition, respectively. These values were markedly higher than those recorded for most of the remaining fractions, suggesting that the compounds responsible for the observed activity were concentrated primarily in these early-eluting fractions. Fraction 8 also demonstrated relatively strong inhibitory activity (50.01 ± 3.38%), indicating that the bioactive constituents were not confined to a single chromatographic fraction but were distributed across multiple fractions.

The analysis of the results presented in Figure 4 shows a moderate inhibition for fractions 4–7, with values ranging from approximately 17% to 24%, whereas fraction 1 showed only weak activity (4.37 ± 1.89%). Interestingly, the inhibitory potential of the total extract (10.32 ± 3.53%) was considerably lower than that of the most active fractions. This observation indicates that CPC fractionation effectively concentrated the compounds responsible for AChE inhibition, which were originally diluted in the crude extract matrix. Such behaviour is commonly observed in bioactivity-guided fractionation of plant extracts, where chromatographic separation allows enrichment of active constituents while reducing interference from inactive components.

Fractions 2 and 3 demonstrated pronounced AChE inhibitory activity. Considering that the observed inhibition was comparable to that of the reference inhibitor, these fractions may contain bioactive constituents responsible for the AChE inhibitory effect of the *P. nudicaule* extract. The phytochemical and spectrometric analysis of the active fractions revealed that the main component of the most active one, namely fraction 3, is amurensinine. The IC_50_ value of this fraction against AChE was determined as 3.08 mg/mL (9.47 mM, expressed as amurensinine equivalents), whereas berberine, used as a positive control, an IC_50_ value of 0.84 mg/mL (2.26 mM) in this assay (see Figures S4 and S5 in the Supplementary File).

### 2.4. Preparative HPLC in the Purification of Amurensinine-Rich Fraction

The optimised chromatographic conditions enabled the effective isolation of the dominant constituent, amurensinine, from the most active fraction. Semi-preparative HPLC separation was monitored at three wavelengths: 254, 290, and 320 nm, allowing precise tracking of the elution of the target compound. The major peak corresponding to amurensinine was collected at 40 min (Figure S3) with high purity, as confirmed by PDA peak purity analysis (Figure S3). The isolated compound was characterized using high-resolution MS and MS/MS data and its structure was further confirmed by NMR spectroscopy (as described in the Section 2.5). Structural assignment by HPLC-MS was based on a detailed analysis of the observed fragmentation pathway, which was discussed in detail in Section 2.1 and presented in Figure 2.

Amurensinine was subsequently evaluated in the AChE inhibition assay and showed a clear dose-dependent effect. At a concentration of 1 mg/mL, corresponding to that used for berberine, amurensinine exhibited 28.2% inhibition (Figure 4B), while berberine showed the inhibitory potential of 54.14%.

### 2.5. NMR-Based Structure Elucidation of Amurensinine

The complete NMR data set, including 1D ^1^H and ^13^C spectra as well as 2D ^1^H−^1^H DQF-COSY, ^1^H−^13^C HSQC, ^1^H−^13^C HMBC, and ^1^H−^1^H NOESY experiments, enabled an unambiguous structural assignment of the investigated compound. The observed pattern of aromatic, methoxy, N-methyl, acetal, and aliphatic resonances was consistent with an aporphine-type alkaloid framework and allowed the compound to be identified as amurensinine.

A key starting point for the assignment was the acetal carbon C2′, which displayed a characteristic and highly diagnostic ^13^C chemical shift at δ_C_ 100.76 ppm. This resonance was unique within the molecule and, through the HSQC spectrum, enabled the identification of the corresponding acetal protons H2′a and H2′b at δ_H_ 5.870 and 5.941 ppm, respectively. The presence of this acetal fragment provided a convenient anchor for tracing the local connectivity pattern by means of long-range heteronuclear correlations.

The differentiation between amurensinine and the closely related structural isomer reframine was based primarily on diagnostic HMBC correlations involving the acetal protons. Both H2′a and H2′b showed long-range correlations to the carbon resonances assigned to C2/C3. Importantly, the same C2/C3 carbon resonances also displayed HMBC correlations with the aromatic protons H1 and H4. This correlation pattern established the position of the acetal-containing moiety relative to the aromatic portion of the molecule and supported the connectivity expected for amurensinine rather than reframine.

Further refinement of the aromatic assignment was achieved by distinguishing H1 and H4 from the remaining aromatic protons, H7 and H10. This step was possible due to characteristic HMBC correlations between H4 and C5 and between H1 and C12. H7 produced diagnostic couplings to C5 and C8, whereas H10 displayed crucial correlations to C9 and C11. These cross-peaks linked the respective aromatic protons to the adjacent aliphatic region of the alkaloid skeleton, thereby resolving the otherwise similar aromatic resonances.

The positions C5 and C12 were differentiated unambiguously based on the HMBC correlation between the N-methyl protons and C12. This correlation provided a decisive reference point for assigning the nitrogen-containing part of the molecule and completed the mapping of the aliphatic fragment. The combined HSQC and HMBC information, supported by the proton-proton connectivities observed in the DQF-COSY spectrum and the spatial information from NOESY, therefore confirmed the full resonance assignment and the identity of the compound as amurensinine.

**Figure 5:**
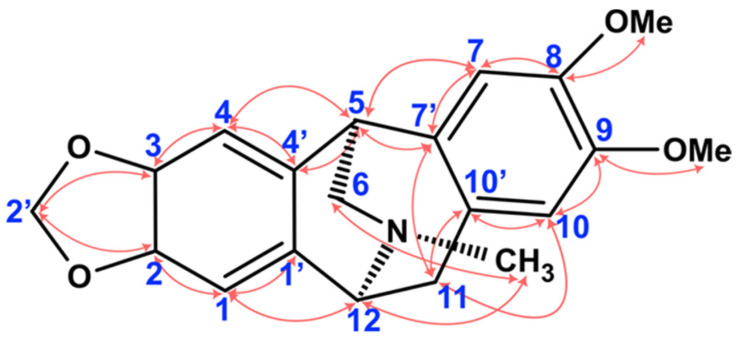
NMR-derived structure of amurensinine. Diagnostic ^1^H−^13^C HMBC correlations are depicted as bidirectional arrows.

The resolved structure of amurensinine, along with the most important HMBC correlations, was presented as Figure 5. The resulting ^1^H and ^13^C chemical shifts and the corresponding spectra are presented in the Supplementary File (Tables S3 and S4, Figures S6 and S7).

The phytochemical and spectrometric analysis of the active fractions revealed that the main component of the most active one, namely fraction 3, is amurensinine.

Isoquinoline alkaloids constitute one of the most extensively studied groups of natural AChE inhibitors and are considered important lead structures in the search for new therapeutic agents for neurodegenerative diseases. Numerous representatives of this class exhibit significant inhibitory activity against AChE, often within the low micromolar range. For example, protoberberine alkaloids such as berberine, coptisine, palmatine, and jatrorrhizine have been reported to exhibit notable inhibitory activity against AChE, with IC_50_ values typically ranging from 0.4 to 5 µM, depending on the compound and assay conditions [51]. Their inhibitory potential is attributed to the presence of a positively charged isoquinoline nitrogen atom and an extended conjugated aromatic system, which enables effective interactions with the enzyme’s catalytic and peripheral sites. Structural studies indicate that the conjugated aromatic B ring plays a crucial role in enzyme binding, whereas hydrogenation of this ring, as observed in dihydroberberine, markedly reduces inhibitory activity. Other isoquinoline alkaloids also demonstrate remarkable potency against cholinesterases. For instance, sanguinine isolated from *Galanthus woronowii* showed stronger AChE inhibition than the clinically used drug galanthamine, with reported IC_50_ values of approximately 0.10–0.30 µM [52,53]. Several studies emphasise the importance of structural features such as aromatic substitution patterns, hydroxyl or methoxy groups, and nitrogen-containing heterocycles in determining cholinesterase inhibitory potency.

In the context of the present study, the relatively strong AChE inhibitory activity observed for selected CPC fractions of *P. nudicaule* may be associated with the presence of isoquinoline-type alkaloids typical for the Papaveraceae family. Fractionation by CPC likely concentrated compounds with structural features favourable for enzyme binding, resulting in increased inhibitory activity compared to the crude extract. It is known that AD is a multifactorial neurodegenerative disorder characterised by a highly complex pathophysiology involving cholinergic dysfunction, amyloid-β aggregation, tau hyperphosphorylation, oxidative stress, neuroinflammation, and synaptic degeneration. However, at the early stages of compound evaluation, preliminary screening studies remain essential; initial investigations are typically focused on single-target interactions, particularly the inhibition of AChE or butyrylcholinesterase (BuChE), and serve as rapid and reliable filters for prioritising biologically relevant candidates. Such screening allows the selection of promising compounds for subsequent, more advanced analyses. Considering that numerous isoquinoline alkaloids reported in the literature exhibit potent cholinesterase inhibition and favourable pharmacological profiles [54], the observed activity of the obtained fractions supports the potential of *P. nudicaule* as a promising natural source of bioactive alkaloids with possible applications in the development of novel therapeutic agents for AD and other dementia-related disorders.

### 2.6. Preliminary Evaluation of the Safety Profile of the Selected Fractions on Human Neuronal Cells

*P. nudicaule* extract and amurensinine were subsequently subjected to a preliminary safety evaluation by assessing their in vitro toxicity toward human neuronal cells using the SH-SY5Y neuroblastoma cell line. Cytotoxicity assays were performed on undifferentiated SH-SY5Y cells. Although differentiated cells more closely resemble mature neurons, undifferentiated SH-SY5Y cells are widely used for initial neurotoxicity screening of plant extracts and provide a sensitive readout due to their high proliferative activity and metabolic demand [55,56].

**Figure 6:**
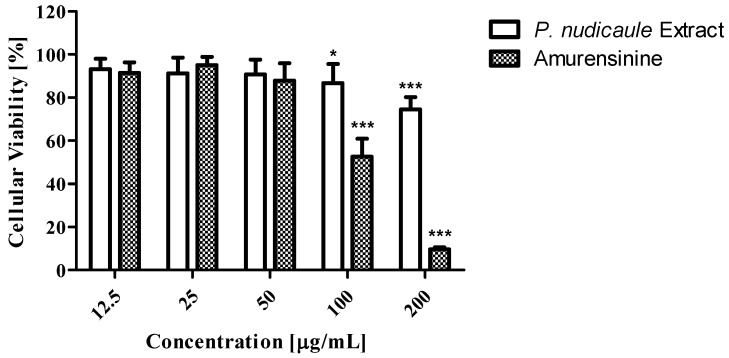
Cytotoxicity of *P. nudicaule* extract and amurensinine towards human neuronal cells SH-SY5Y following 24h incubation; histogram shows mean cellular viability ± SD; *n* = 3, * *p* < 0.05; *** *p* < 0.001.

The performed analysis demonstrated that the total extract did not exhibit significant toxicity toward the analysed cells within the tested concentration range of 12.5–200 μg/mL. Amurensinine reduced cell viability (<50%) at concentrations of 100 and 200 μg/mL (see Figure 6).

In relation to the observed cytotoxic effects of amurensinine, it is important to consider commonly used benchmarks for in vitro cytotoxicity. A decrease in SH-SY5Y cell viability was observed at concentrations of 100–200 µg/mL, corresponding to approximately 296 µM for amurensinine. According to widely cited criteria for anticancer drug screening supported by the U.S. National Cancer Institute (NCI), compounds with IC_50_ values above 50 µM are generally considered to exhibit low or no significant cytotoxic activity [57].

Similarly, classical guidelines proposed by Kurt Hostettmann and co-workers classify crude plant extracts as cytotoxic when IC_50_ values are ≤100 µg/mL, whereas higher values are regarded as inactive; however, these thresholds are less stringent for pure compounds. In this context, the cytotoxic effect observed for amurensinine at ≥100 µg/mL should be interpreted as relatively weak [58].

Importantly, no significant reduction in cell viability was observed at lower concentrations, suggesting a potential therapeutic window in which the compound may exert biological activity without causing pronounced cytotoxicity. Moreover, to the best of our knowledge, this is the first study to evaluate the effects of amurensinine on human neuronal cells, underscoring the preliminary nature of these findings and the need for more detailed dose–response and mechanistic studies.

A limitation of the present study is the use of undifferentiated SH-SY5Y cells, which do not fully recapitulate the mature neuronal phenotype. Validation in differentiated SH-SY5Y cells, primary neuronal cultures, or organotypic models would further strengthen the safety profile and is planned for future studies.

## 3. Materials and Methods

### 3.1. Plant Material and Extraction

The aerial parts of *P. nudicaule* were collected from the Gorkhi-Terelj National Park (Erdene Soum, Tuv Province, Mongolia) during the peak of its blooming season (late July). The botanical identity of the plant has been confirmed, and the voucher specimens have been deposited in the Herbarium of the School of Pharmacy at the Mongolian National University of Medical Sciences (MNUMS). After collection, herbal samples were dried and milled. Then, 320 g of plant material was subjected to maceration process in glass bottles for 7 days using pure methanol as an extraction solvent. Every day the extract was additionally sonicated for 1 h. Macerates were combined and evaporated to dryness (45 °C) using rotary evaporator (Neuberger, D-79112, Rothenburg, Germany).

### 3.2. Chemicals and Reagents

HPLC-grade solvents (ethanol, butanol, *n*-hexane, ethyl acetate, chloroform, acetonitrile, and formic acid) used for the CPC procedure and HPLC analysis were manufactured by Avantor Performance Materials (Gliwice, Poland). Reagents of spectroscopic purity (acetonitrile, formic acid, and water) were purchased from Sigma–Aldrich (Steinheim, Germany). Deionised water (>18 mω) was obtained using a water purification system (Millipore, Molsheim, France). The reference standard of the alkaloid berberine (purity > 95%) used in the bioassay was provided by Sigma–Aldrich (Steinheim, Germany).

### 3.3. HPLC-ESI-QTOF-MS/MS Analysis

The qualitative analysis of *P. nudicaule* extract and isolated alkaloid was performed using a 1290 Infinity HPLC chromatograph (Agilent Technologies, Santa Clara, CA, USA) hyphenated to a 6530B Accurate-Mass Quadrupole Time-of-Flight (Q-ToF) mass spectrometer equipped with an electrospray (ESI) ioniser. The separation of bioactive compounds was carried out on a Zorbax Eclipse Plus RP-18 chromatographic column (150 mm × 2.1 mm; I.D., dp 3.5 μm; Agilent Technologies, Santa Clara, CA, USA) at 25 °C. A gradient of water (A) and acetonitrile (B), both containing addition of 0.1% (*v*/*v*) formic acid, was pumped at the flow rate of 0.25 mL/min as follows: 0–10 min/0–20% B, 10–15 min/20–40% B, isocratic run from 17 min to 22 min with 95% of B in A, and back to 1% of B (24–40 min). The UV spectra were recorded at 254 and 290nm. The mass spectra were acquired on the freshly calibrated instrument in two ionisation modes, using full-scan acquisition over the *m*/*z* range 40–1200. The MS/MS spectra were recorded out of the simultaneous fragmentation of two most intensive *m*/*z* signals in a scan. The following spectroscopic parameters were applied: gas and sheath gas flows, 12 L/min; gas temperature, 250 °C; sheath gas temperature, 300 °C; nebulizer gas pressure, 35 psig; skimmer voltage, 65 V; Vcap, 3000 V; nozzle voltage, 1000 V; fragmentor voltage, 110 V and collision-induced dissociation (CIDs) energies set at 10 and 20 eV. For data acquisition and compounds analysis MassHunter software (version 10.0) was used. The chemical structures of the compounds were identified based on the obtained UV spectra, retention times, and self-analysis of their fragmentation behaviour, compared with commonly used mass spectral libraries (MassBank (https://massbank.eu/MassBank/ accessed on 30 April 2026), PubChem (https://pubchem.ncbi.nlm.nih.gov/ accessed on 30 April 2026) and the scientific literature.

The identified compounds were annotated according to the Metabolomics Standards Initiative (MSI) guidelines [59,60,61].

### 3.4. Centrifugal Partition Chromatography (CPC)

#### 3.4.1. Selection of a Biphasic Solvent System

The selection of an appropriate biphasic solvent system is a critical step for the effective separation of constituents from the *P. nudicaule* extract. Therefore, several solvent systems were evaluated to achieve efficient fractionation of the analysed extract. Quaternary solvent systems of similar composition were selected to obtain a wide range of partition coefficient (K_D_) values for the constituents present in the extract. Quaternary mixtures composed of two nonpolar and two polar solvents allow convenient control of the overall polarity of the solvent system. They are therefore the first choice selection for the investigated poppy extract.

Based on these considerations, the following solvent systems were investigated: n-hexane/n-BuOH/EtOH/H_2_O (3:12:6:15, *v*/*v*/*v*/*v*); n-hexane/n-BuOH/EtOH/H_2_O (1:14:6:15, *v*/*v*/*v*/*v*); n-hexane/EtOAc/EtOH/H_2_O (5:3:4:4, *v*/*v*/*v*/*v*); and MtBE/n-BuOH/ACN/HCl (2:2:1:5, *v*/*v*/*v*/*v*). Each solvent system was evaluated for its suitability for fractionating the total extract.

For solvent system selection, 4 mL of each biphasic mixture was prepared in separatory funnels. Subsequently, 10 mg of the extract was added and the mixture was shaken for 2 min to allow proper distribution of the constituents between the phases. After phase separation, 1 mL from each phase was filtered and analysed by HPLC-PDA to determine partition coefficient values (K_D_) (see Supplementary File Table S2).

#### 3.4.2. Determination of K_D_ Using HPLC-PDA Method

The determination of the partition coefficient values (K_D_) for active constituents of *P. nudicaule* was performed on a Shimadzu HPLC system (Shimadzu, Tokyo, Japan) equipped with an autosampler (SIL-20A HT), an automatic degasser (DGU-20A 3R) and a DAD detector (SPD-M20A) using an RP-18 chromatographic column Supelco 250 mm × 4.0 mm, pore diameter: 5 μm by Sigma Aldrich (St. Louis, MO, USA). A gradient of acetonitrile (B) and (A) water with the addition of 0.1% formic acid to both eluents was used as mobile phase at a flow rate of 1 mL/min and changed according to the following scheme: 0–40 min a linear grade up to 20% of B; 40–60 min/40% of B; in 62min isocratic run with 1% B in A. A 10 μL aliquot of the sample was injected onto the chromatographic column. The total run was set to 70 min, the postrun to 10 min, and the temperature to 25 °C. The UV spectra were recorded at 290 nm. The K_D_ values calculated for each peak were expressed as the peak area of target compounds in the stationary phase divided by their areas in the mobile phase.

#### 3.4.3. Fractionation of the *P. nudicaule* Extract by CPC

The CPC-based fractionation of *P. nudicaule* extract was performed using Armen SCPC-250-L chromatograph (Armen, Saint Ave, France) equipped with a 250 mL rotor, a UV detector (Flesh06S DAD 600) and a fraction collector (LS-5600). The biphasic solvent system, chosen for the isolation of the target compound from the methanolic extract of *P. nudicaule*, consisting of n-hexane–n-butanol–ethanol–water (3:12:6:15; *v*/*v*/*v*/*v*), was prepared before use. The CPC process was started by filling, the slowly rotating column (500 rpm), with upper stationary phase with a flow rate of 20 mL/min for 15 min. Then, 500 mg of extract was dissolved in the smallest possible volume (4 mL) of a 1:1 (*v*/*v*) mixture of lower and upper phases and injected into the system. The elution was carried out with the lower phase as the mobile phase in descending mode, with rotation set at 1200 rpm and a flow rate of 4 mL/min. After 1 h, extrusion was performed using fresh portions of the upper phase for the next 70 min at the same rotation speed and flow rate. All 30 fractions (8 mL each) were collected. Detection of compounds was carried out at two wavelengths: 254 nm and 290 nm (for alkaloids). The whole procedure was repeated two times.

The fractions of interest were condensed using an Eppendorf Concentrator evaporator (Hamburg, Germany), dissolved in methanol, filtered (nylon syringe filter, 0.1 µm, LAB-EX KFT, Budapest, Hungary) and subjected to HPLC-MS analysis in the formerly described conditions.

### 3.5. Preparative HPLC in the Final Purification of Amurensinine

In the next step, following CPC fractionation, the fraction 3 enriched in amurensinine was subjected to purification using a preparative HPLC system comprising an LC-20AP preparative liquid chromatograph, an SPD-M40 DAD detector, and an LH-40 liquid handler. Briefly, 100 mg of the sample was dissolved in 1mL of 20% methanol (*v*/*v*) and injected on ReproSil-Pur 120 Si chromatographic C-18-AQ column (150 × 20 mm, 5 µm id) supplied by Dr Maisch (Ammerbuch, Germany).

For chromatographic separation, the mobile phase composed of: solvent A consisting of 0.1% of formic acid in water (*v*/*v*) and solvent B composed of 0.1% of formic acid in acetonitrile (*v*/*v*) was prepared. Elution was performed at a flow rate of 19 mL/min, using a gradient of solvent B in A as follows: 1% B at 0 min, 20% B/40 min, 40% B/60 min, 95% B/62–67min and 1% B till 80 min. The column temperature was set at 26 °C, and the chromatographic profile was monitored at three wavelengths: 254, 290, and 320 nm. The recorded chromatogram was presented in the Supplementary File (Figure S3). During the evaporation process, the sample was rinsed with water to remove residual formic acid.

### 3.6. NMR-Based Structure Elucidation of Amurensinine

NMR measurements were performed in DMSO-d_6_ at 25 °C on a 700 MHz NMR spectrometer equipped with a QCI-CryoProbe at the Institute of Bioorganic Chemistry, Polish Academy of Sciences, Poznań, Poland. The acquired data set comprised one-dimensional ^1^H and ^13^C NMR spectra, together with two-dimensional ^1^H−^1^H DQF-COSY, ^1^H−^13^C HSQC, ^1^H−^13^C HMBC, and ^1^H−^1^H NOESY experiments. Chemical shifts were referenced to the residual solvent resonance of DMSO-d_6_ (δ_ref_ = 2.500 ppm) and are reported in ppm.

The ^1^H 90° pulse length was 7.14 μs. For all proton-detected experiments, the spectral width in the ^1^H dimension was 7716 Hz. The spectral widths in the indirect ^13^C dimension were 29,177 Hz for HSQC and 42,277 Hz for HMBC. The DQF-COSY, NOESY, HSQC, and HMBC spectra were acquired with 112, 64, 16, and 112 scans, respectively, using data matrices of 2048 × 256 points. All two-dimensional spectra were processed to a final matrix size of 2K × 1K.

### 3.7. In Vitro Biological Activity Determination Towards the Acetylcholinesterase Inhibitory Potential

The AChE inhibitory activity was evaluated using a Fluorometric Acetylcholinesterase Inhibition Assay (FAIA) performed on a Greiner CELLSTAR^®^ 96-well black microplate. Fluorescence measurements were carried out using a GloMax^®^ Explorer microplate reader (Promega Corporation, Madison, WI, USA). Reagents used for the fluorescence assay included AChE from *Electrophorus electricus* (electric eel), Trizma base, bovine serum albumin (BSA), 4-methylumbelliferyl acetate (4-MUA), berberine chloride, all purchased from Sigma-Aldrich (St. Louis, MO, USA). Dimethyl sulfoxide (DMSO) and ethanol were of analytical reagent grade. Redistilled water was used throughout the experiments.

The enzyme solution was prepared at a concentration of 3 U/mL by diluting the stock solution (50 U/mL) with the experimental buffer. The buffer was prepared by dissolving Trizma base and bovine serum albumin in distilled water, followed by adjustment of the pH to 7.8. The substrate solution (4-methylumbelliferone acetate, 1.5 mg/mL) was prepared by dissolving the compound in a mixture of ethanol and water (2:1, *v*/*v*).

The total extract and CPC fractions obtained from *Papaver nudicaule* herb were dissolved in dimethyl sulfoxide (DMSO) at a concentration of 5 mg/mL. Berberine was selected as the reference inhibitor in this assay due to its well-documented AChE inhibitory activity and a structure resembling the herein described class of alkaloids present in *P. nudicaule*, moreover it provided consistent and reproducible results during method optimization and preliminary experiments [62].

4-Methylumbelliferyl acetate was used as a fluorogenic substrate in accordance with the validated FAIA protocol. The substrate enables sensitive fluorescence-based detection of AChE activity through the formation of fluorescent 4-methylumbelliferone.

Berberine solution (a purity > 95%) (BER) was prepared in DMSO and tested at a concentration of 1 mg/mL for comparison with the investigated fractions. During the assay, 10 µL of the test samples or DMSO was mixed with 180 µL of the enzyme solution and incubated for 25 min at 37 °C. Then, 10 uL of 4-MU solution was added, and the reaction mixture was incubated for an additional 20 min. After this step, the measurement was taken at excitation 365 nm and emission 450 nm. To account for any potential effect of DMSO on basal AChE activity, solvent control wells containing the same final concentration of DMSO but no test sample were included in each experiment. The final concentrations in the microplate wells were 0.5 mg/mL for the extract and fractions and 0.05 mg/mL for BER.

The percentage of AChE inhibition was calculated according to the following equation: % Inhibition = [(F_control_ − F_sample_)/F_control_] × 100, where F_control_ represents the fluorescence intensity of the control reaction containing DMSO and F_sample_ represents the fluorescence intensity measured in the presence of the tested sample. The fluorescence values for the tested samples were corrected by subtracting the background signal. The background was determined using the same volume of the tested fraction, extract, or pure compound mixed with buffer, resulting in a total volume identical to that of the corresponding assay samples. The fluorescence signals were recorded and processed in Microsoft Excel. All samples were applied in triplicate. Differences between control sample (DMSO) and tested samples were analyzed using one-way ANOVA followed by Tukey’s HSD post hoc test. Results were considered statistically significant at *p* < 0.05. Statistical analyses were performed using GraphPad Prism 9 (GraphPad Software, Boston, MA, USA).

### 3.8. In Vitro Cytotoxicity Towards Human Neuronal Cells

*P. nudicaule* extract and the isolated compound amurensinine were evaluated for their potential in vitro cytotoxicity against the human neuroblastoma cell line SH-SY5Y (ATCC CRL-2266, LGC Standards, Łomianki, Poland), which is widely used as an in vitro model of human neurons [55,56]. Cells were cultured in Dulbecco’s Modified Eagle Medium/Nutrient Mixture F-12 (DMEM/F12; Sigma-Aldrich, USA) supplemented with 10% fetal bovine serum (FBS, Pan Biotech, Aidenbach, Germany) and 1% penicillin/streptomycin under standard culture conditions (37 °C, 5% CO_2_).

For the experiment, 1 × 10^4^ cells were seeded into each well of a 96-well plate. Following overnight incubation, the cells were treated with the extract or isolated amurensinine at concentrations ranging from 12.5 to 200 μg/mL. Control cells were cultured in the presence of an equivalent volume of the solvent (DMSO, Honeywell International Inc., Phoenix, AZ, USA). After 24 h of treatment, 10 μL of a 0.25 mg/mL resazurin solution (Sigma-Aldrich, St. Louis, MO, USA) was added to each well, and the cells were incubated for an additional 3 h. Fluorescence was subsequently measured using a microplate reader (Molecular Devices, San Jose, CA, USA) at excitation/emission wavelengths of 540/590 nm. Cell viability was expressed as a percentage relative to the control cells. The cytotoxicity study was performed in three independent experiments, each conducted with five technical replicates per condition. Results are presented as mean ± standard deviation (SD). Differences between control cells and cells treated with different concentrations of the tested samples were analyzed using one-way ANOVA followed by Tukey’s HSD post hoc test. Results were considered statistically significant at *p* < 0.05. Statistical analyses were performed using GraphPad Prism 9 (GraphPad Software, Boston, MA, USA).

## 4. Conclusions

This study provides an in-depth phytochemical and bioactivity-oriented characterisation of *Papaver nudicaule*, demonstrating that its chemical composition has been comprehensively elucidated. The applied HPLC–ESI–QTOF-MS/MS approach enabled the tentative identification of 34 metabolites, confirming that the species is a rich source of structurally diverse isoquinoline alkaloids alongside flavonoid derivatives. The pronounced diversity of alkaloid structures highlights a marked chemical potential of this plant. Thanks to the application of centrifugal partition chromatography (CPC), which was found to be highly effective for fractionating its alkaloid-rich extract, selective enrichment of metabolites was achieved without the drawbacks associated with solid stationary phases. The obtained fractions allowed a clear differentiation of biological activity profiles during the in vitro assessment of AChE inhibitory potential.

Importantly, the selected fractions exhibited a marked AChE inhibitory activity. The high activity of the most potent fraction was attributed to amurensinine, suggesting that isopavine-type alkaloids may play a key role in enzyme inhibition. These findings are particularly relevant in the current context of an increasing demand for novel and effective agents capable of delaying the progression of neurodegenerative disorders. Inhibition of AChE remains one of the primary therapeutic strategies in the management of cognitive impairment and memory disorders, and a significant proportion of currently used drugs are of natural origin or are inspired by plant-derived compounds. Therefore, the demonstrated activity of amurensinine provides important evidence supporting the potential of isopavine alkaloids as promising lead structures in the development of new therapeutics targeting dementia-related diseases.

Overall, the amurensinine isolated from the total extract exhibited only weak cytotoxic effects in human neuronal cells, observed exclusively at relatively high concentrations. At the same time, no toxicity was detected at lower doses. These findings indicate a preliminary safety profile and support further investigation of this compound at non-cytotoxic concentrations. *P. nudicaule* emerges as a plant with a highly promising chemical profile and remarkable alkaloid diversity, supporting its potential as a valuable source of bioactive compounds. These findings provide a strong basis for further targeted isolation, structure–activity studies, and pharmacological evaluation of individual constituents in the context of neurodegenerative diseases.

## Figures and Tables

**Table 1:** Compounds tentatively identified in *P. nudicaule* extract by HPLC-ESI-QTOF-MS/MS analysis in the positive and negative ion mode (CID—collision induced energy), Rf—retention factor.

No	Rf (min)	Tentative Identification	Molecular Formula	Experimental *m*/*z* (Error in ppm)	MS/MS CID 20 eV	Ref.
1	12.07	Higenamine-*O*-glucoside	C_22_H_28_NO_8_^+ a^	434.1811 (0.36)	272.1276; 255.1010; 161.0597; 107.0489	[20]
2	13.60	Norcoclaurine (=higenamine= Demethylcoclaurine)	C_16_H_17_NO_3_ ^a^	272.1274 (−2.66)	**255.1008**; 161.0587; 143.0479; 107.0490	[21]
3	13.75	8,14-Dihydroflavinantine	C_19_H_23_NO_4_ ^a^	330.1692 (−2.38)	**315.1411;** **285.1113**; 194.1167; 175.0746	[22]
4	14.51	Kaempferol 3-sophoroside-7-glucoside	C_33_H_40_O_21_ ^b^	771.1941 (−6.21)	609.1415; 446.0811; 285.0366; 284.0303	[10]
5	14.72	Thalidicine/thalidine	C_19_H_21_NO_4_ ^a^	328.1538 (1.63)	**297.1113**; 285.1110; 265.0921; 251.0701; 237.0904; 192.1010; 178.0852	Fragmentation; [23]
6	15.15	Magnocurarine	C_19_H_23_NO_3_ ^a^	314.1741 (−6.29)	299.0960; **269.1003**; 237.0759; 175.0645; 145.0542; 137.0509; 121.0557; **107.0425**; 58.0622	[24,25]
7	15.17	Not assigned	n.d.	318.1897 ^a^	289.1633; 275.1483; **257.1391**; 206.1056; 189.0804; **177.0808;** 165.0899; 151.0747	
8	15.72	Coclaurine/Isococlaurine	C_17_H_19_NO_3_ ^a^	286.1438 (0.11)	269.1168; 237.0906; 175.0746; 145.0464 137.0593; **107.0490**	PubChem
9	15.95	N-methylcoclaurine/ N-methylisococlaurine	C_18_H_21_NO_3_ ^a^	300.1596 (0.6)	**269.1168**; 237.0905; 209.0945; 192.1003; 175.0748; 145.0641; 137.0594; **107.0489**	[26]
10	16.24	Caffeic acid	C_9_H_8_O_4_ ^b^	179.0343 (−3.79)	**135.0444**	[27]
11	16.47	2-Methylmagnocurarine	C_20_H_26_NO_3_^+ a^	328.1900 (−2.2)	**283.1073**; 252.0916; 189.0735; 151.0612; 121.0537; **107.0394**; **58.0607**	[25]
12	16.73	Thalisopavine	C_20_H_23_NO_4_ ^a^	342.1700 (0.04)	**311.1505**; 299.1494; 280.1083; 265.1047; 251.1243; 208.1287	Fragmentation; [23]
13	16.83	Reticuline	C_19_H_23_NO_4_ ^a^	330.1698 (−0.56)	299.1267; **192.1015**; 175.0745; 143.0486; 137.0591; 107.0495	[21,28]
14	17.00	Not assigned (Isomer of compound 7)	n.d.	318.2057 ^a^	289.1791; **257.1531**; 206.1172; 189.0904; **177.0905;** 165.0899; 151.0747	
15	17.67	Amurensine isomer 1	C_19_H_19_NO_4_ ^a^	326.1396 (2.82)	**295.0972**; 283.0969; 263.0703; 237.0910; 205.0646; 178.0849	Fragmentation
16	17.73	Quercetin-O-gentiobioside	C_27_H_30_O_17_	625.1430 (3.16)	463.0879; 300.0271; 178.9954	PubChem
17	18.05	Amurensine Isomer 2	C_19_H_19_NO_4_ ^a^	326.1390 (0.97)	309.1114; 295.0959; 283.0964; 263.0690; 178.0860; 163.0623; 151.0749; 119.0486	[25]
18	18.25	Methylthalisopavine	C_21_H_25_NO_4_ ^a^	356.1857 (0.18)	**325.1429;** 313.1427; 294.1240; 279.1006; 263.1041; 251.1061	[23]
19	18.26	Sophoraflavonoloside = Kaempferol 3-*O*-β-sophoroside	C_27_H_30_O_16_ ^b^	609.1434 (−4.44)	429.0777; **284.0268;** 255.0245	[10]; Pubchem
20	18.54	Gossypitrin	C_21_H_20_O_13_ ^b^	479.0827 (−0.86)	**317.0291**; 299.0185	[9]; Pubchem
21	18.88	Amurensinine *	C_20_H_21_NO_4_ ^a^	340.1535 (−2.46)	**309.1122**; 297.1118; 278.0931; 263.0702; 251.1058	PubChem; [8,23,29,30]
22	19.06	Quercetin-*O*-hexoside isomer 1	C_21_H_20_O_12_ ^b^	463.0882 (0)	301.0342; 271.0235; 151.0027	Pubchem
23	19.40	Amuresinine N-oxide	C_20_H_21_NO_5_ ^a^	356.1490 (−0.7)	309.1115; 297.1118; 295.0955; 278.0928; 263.0689; 251.1049	[31]
24	19.45	Muramine	C_22_H_27_NO_5_ ^a^	386.1945 (−4.41)	222.1116; 205.1077; **204.1011**; 190.0853; 181.0851; 165.0899	[25]
25	19.47	Dihydrocryptopine/Demethylatedmuramine	C_21_H_25_NO_5_ ^a^	372.1802 (−0.94)	**354.1694;** 323.1265; 265.1211; 191.0888; **190.0858**; 165.0906; 151.0744	[25,32]
26	19.54	Quercetin-*O*-glucoside isomer 2	C_21_H_20_O_12_ ^b^	463.0900 (3.88)	301.0349	Fragmentation;
27	19.73	α-Allocryptopine	C_21_H_23_NO_5_ ^a^	370.1646 (−0.81)	352.1536; 189.0764; **188.0700**; 181.0853; 165.0903; 149.0590	[32,33]
28	19.80	Reframidine	C_19_H_17_NO_4_ ^a^	324.1219 (−1.04)	**293.0802**; 281.0800; 263.0691; 251.0696; 235.0744; 177.0690	Fragmentation; [23]
29	20.21	Cryptopine	C_21_H_23_NO_5_ ^a^	370.1647 (−1.35)	352.1534; 209.0932; 206.0821; **190.0860**; 188.0701; 181.0852; 161.0593; 149.0591; 135.0437	[32,34]
30	20.68	N-Methylcanadine	C_21_H_24_NO_4_^+ a^	354.1699 (−0.24)	338.1380; 309.1123; 190.0854; 164.0823; 149.0594	[25]
31	20.81	Alborine	C_22_H_22_NO_6_^+ a^	396.1436 (−1.43)	381.1213; 366.0994; 351.0872; 322.1065; 291.0644; 275.0690; 192.1007; 116.003	PubChem; fragmentation; [7]
32	20.98	Berberine	C_20_H_18_NO_4_^+ a^	336.1233 (0.74)	**321.0983**; 320.0906; 306.0760; 304.0958; 292.0957; 278.0817	[18,25]
33	21.60	Luteolin	C_15_H_10_O_6_ ^b^	285.0406 (0.48)	151.0031; **133.0288**; 107.0131	Pubchem
34	21.33	Not assigned	nd	274.2734 ^a^	256.2632; 230.2472; 106.0862; 88.0759	

Abbreviations: ^a^—[M + H]^+^; ^b^—[M − H]^−^; n.d.—not determined, *—confirmed by NMR, bold font in the MS/MS spectra stands for the major *m/z* fragments.

## Data Availability

All data are present in the manuscript and Supplementary file to this manuscript.

## Supplementary Materials

The following supporting information can be downloaded at: https://www.mdpi.com/article/10.3390/molecules31132249/s1, Figure S1: The TIC chromatogram of amurensinine (fraction 3) in the HPLC-MS analysis with the MS spectra for two selected retention times proving its purity; Figure S2: The HPLC-MS fingerprint of the fraction 2 from CPC separation in the negative ion mode used for the acetylcholinesterase inhibition assay. Figure S3: The semi-preparative HPLC chromatogram of fraction 3 from CPC separation, recorded at 290 nm with amurensinine with the retention time of 40 min (fraction 2:21). Figure S4. Concentration-dependent inhibition of AChE by amurensinine-isolated from fraction 3 in FAIA. The calculated IC_50_ value is 3.08 mg/mL. Figure S5: Concentration-dependent inhibition of AChE by berberine standard (positive control) in FAIA. The calculated IC_50_ value is 0.84 mg/mL. Figure S6: The diagnostic region of the edited ^1^H−^13^C HSQC spectrum. Red colour corresponds to the positive-phase cross-peaks, indicating methyl (CH_3_) and methine (CH) groups. The blue colour represents negative-phase correlations, indicating the presence of methylene (CH_2_) groups. Figure S7. The diagnostic region of the edited ^1^H−^13^C HMBC spectrum. Table S1: MS/MS spectra of the tentatively identified compounds recorded in the CID energies of 10 and 20 eV. Table S2: Partition coefficient values obtained for the investigated biphasic solvent systems. Table S3: Assigned ^1^H and ^13^C NMR chemical shifts of amurensinine recorded in DMSO-d_6_ at 25 °C. Table S4: The NMR data obtained for the purified amurensinine.
