# Supplementary material for: Compositional Studies and Bioactivity-Guided Fractionation of Acetylcholinesterase Inhibitors in Papaver nudicaule from Mongolia—The Role of Amurensinine
Source: Molecules. 2026 Jun 26;31(13):2249. doi: 10.3390/molecules31132249 (PMC13362747; doi:10.3390/molecules31132249)
Supplement: Supplementary file 1 [file molecules-31-02249-s001.zip › molecules-4352296-supplementary.pdf]

molecules

MDPI

Table S1. MS/MS spectra of the tentatively identified compounds recorded in the CID energies of 10 and 20 eV

No	Rt (min)	Compound	Molecular formula	*m/z* measured (diff in ppm)	MS/MS spectra (CID 10 and 20)
1.	12.07	Higenamine glucoside	$C_{22}H_{28}NO_8$	434.1811	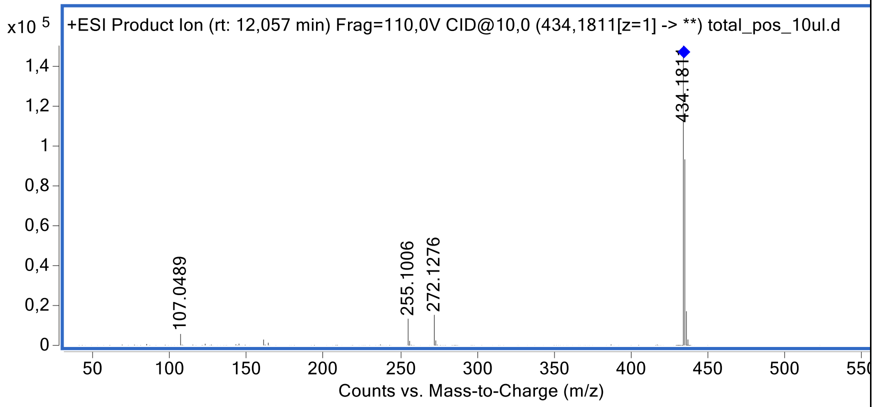 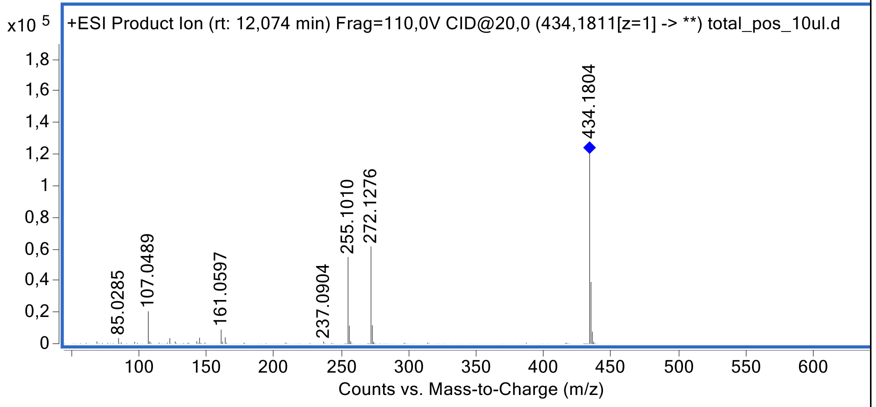

2.	13.60	Norcoclaurine	$C_{16}H_{17}NO_3$	272.1274	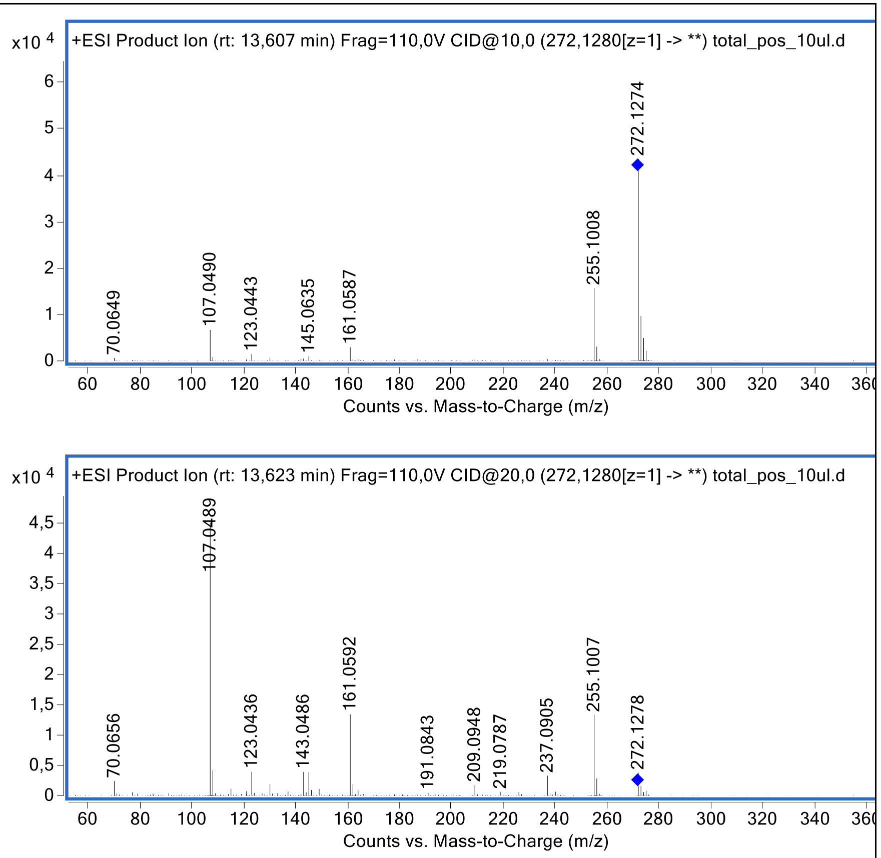

<table>
<tr>
<td>3.</td>
<td>13.75</td>
<td>8,14-dihydroflavinantine</td>
<td>$C_{19}H_{23}NO_4$</td>
<td>330.1692</td>
<td>
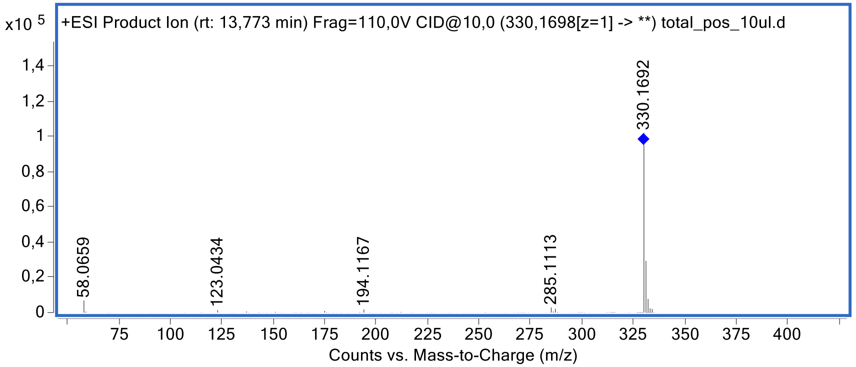

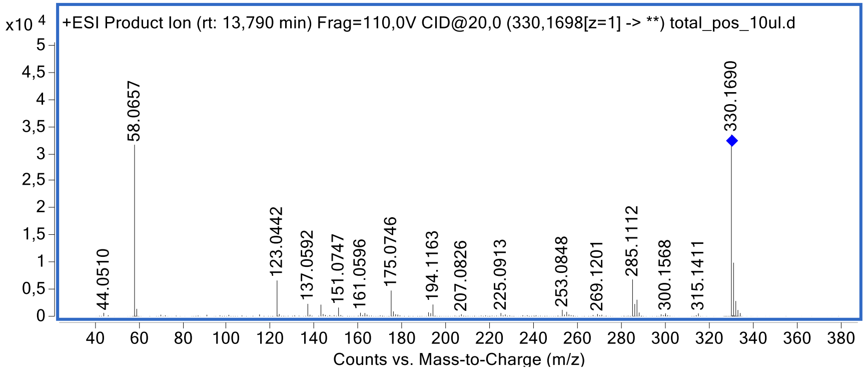

</td>
</tr>
<tr>
<td>4.</td>
<td>14.51</td>
<td>Kaempferol 3-sophoroside-7-glucoside</td>
<td>$C_{33}H_{40}O_{21}$</td>
<td>771.1941</td>
<td>
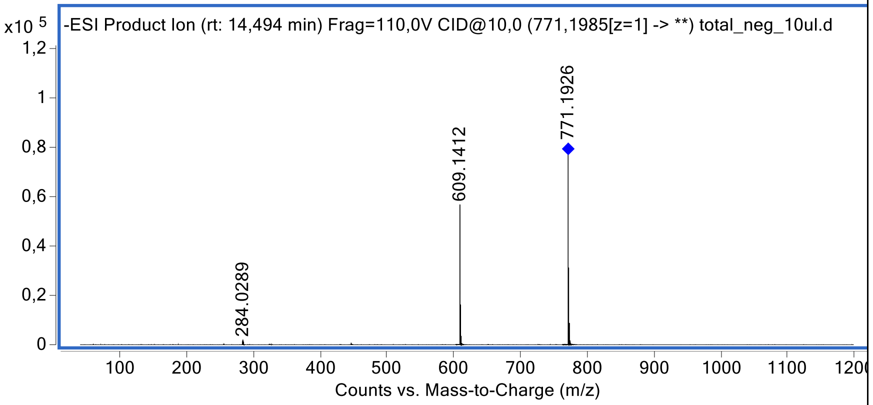

</td>
</tr>
</table>

					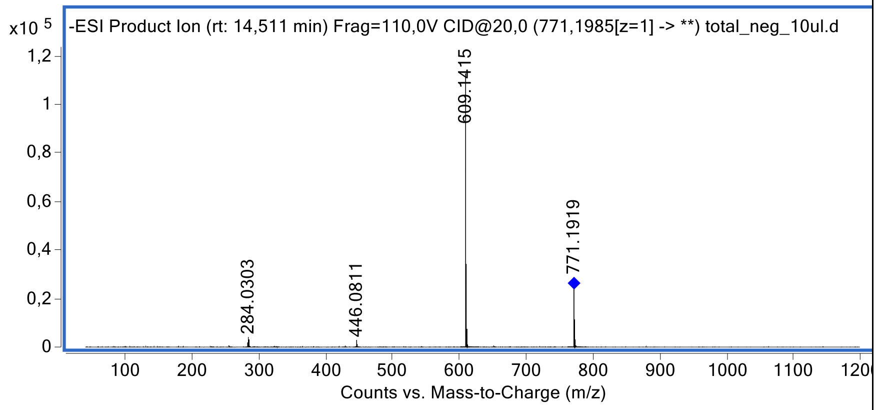
5.	14.72	Thalidicine/thalidine	$C_{19}H_{21}NO_4$	328.1538	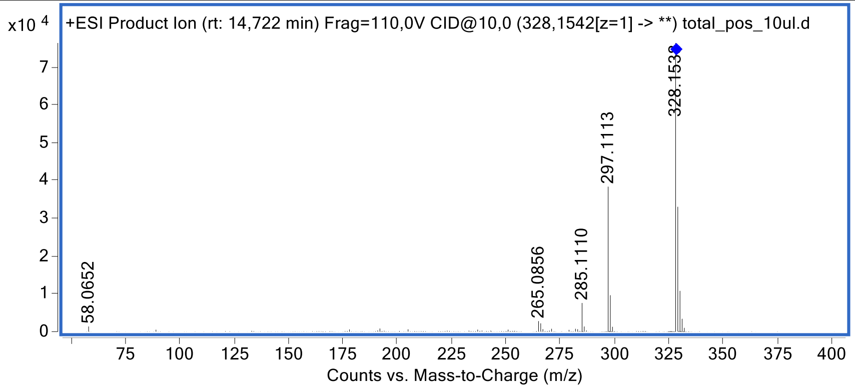 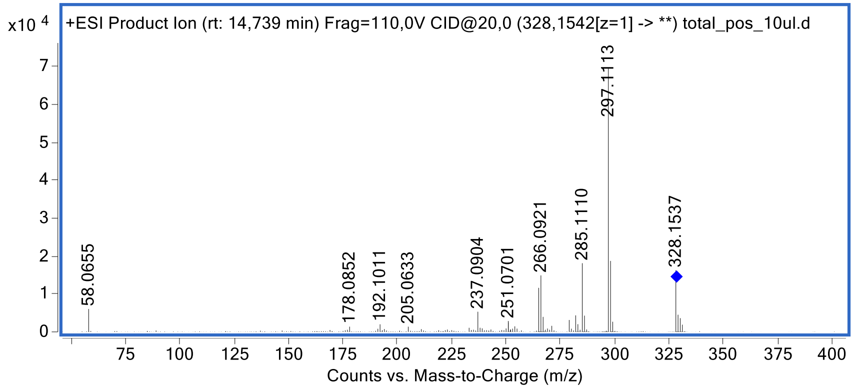

6.	15.15	Magnocurarine	$C_{19}H_{23}NO_3$	314.1741	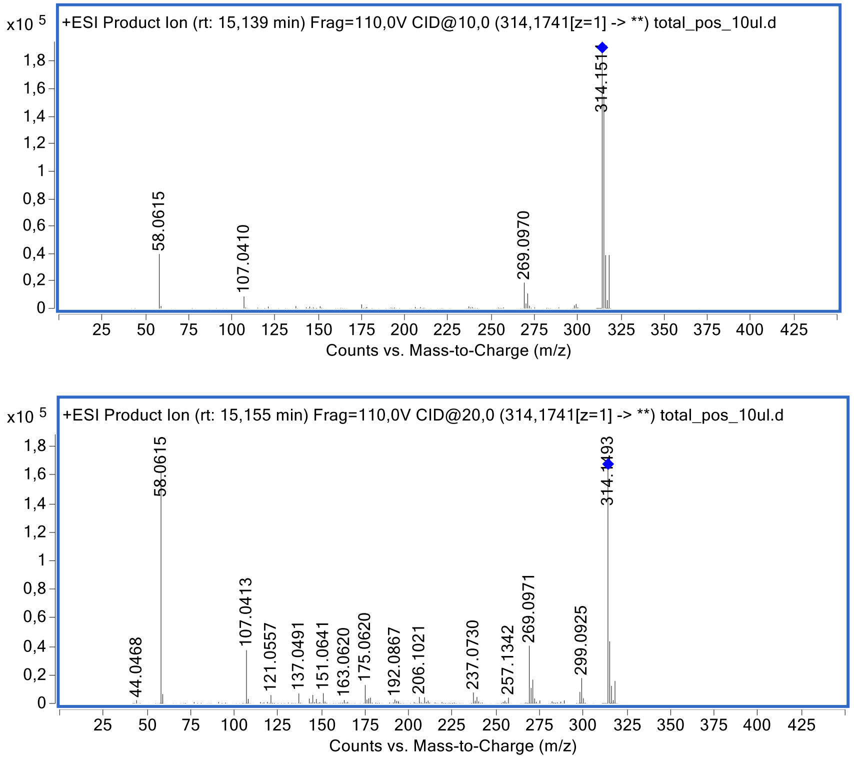

7.	15.17	Not assigned	n.d.	318.1897	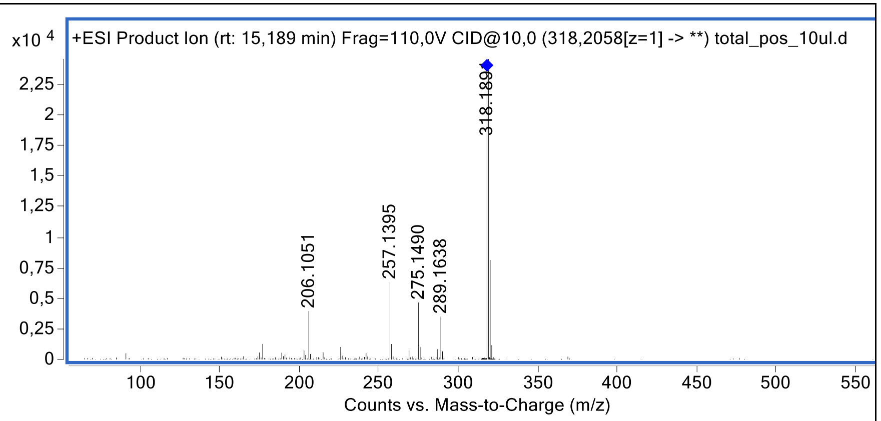 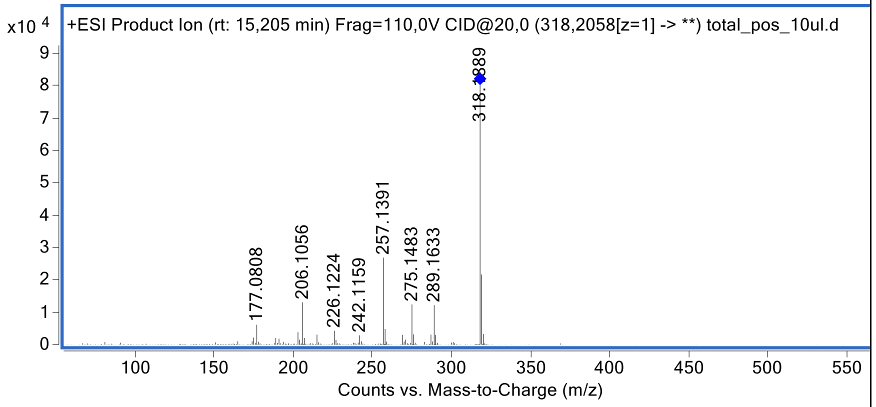

8.	15.72	Isococlaurine or Coclaurine	$C_{17}H_{19}NO_3$	286.1438	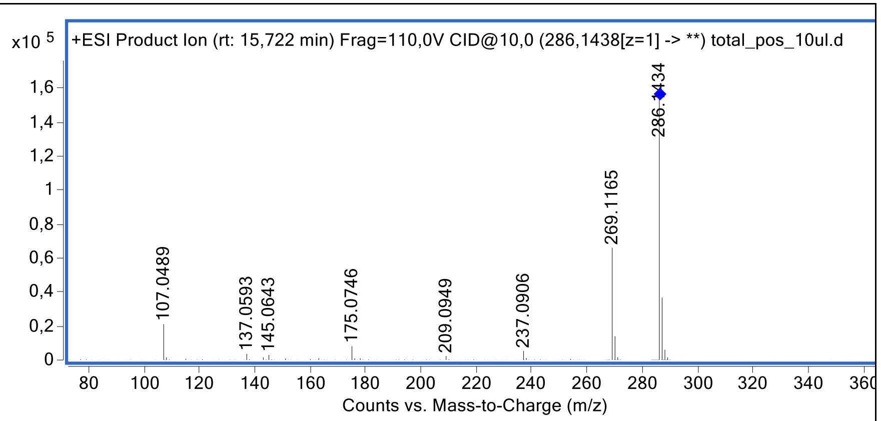<br>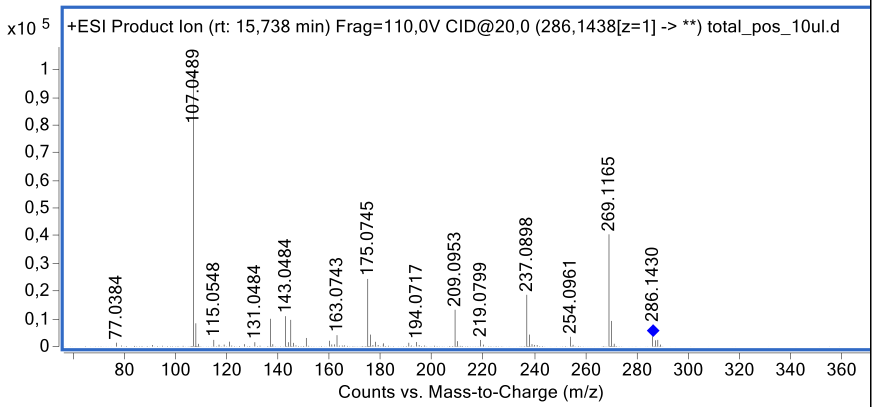

9.	15.955	N-methylcoclaurine or N-methylisococlaurine	$C_{18}H_{21}NO_3$	300.1596	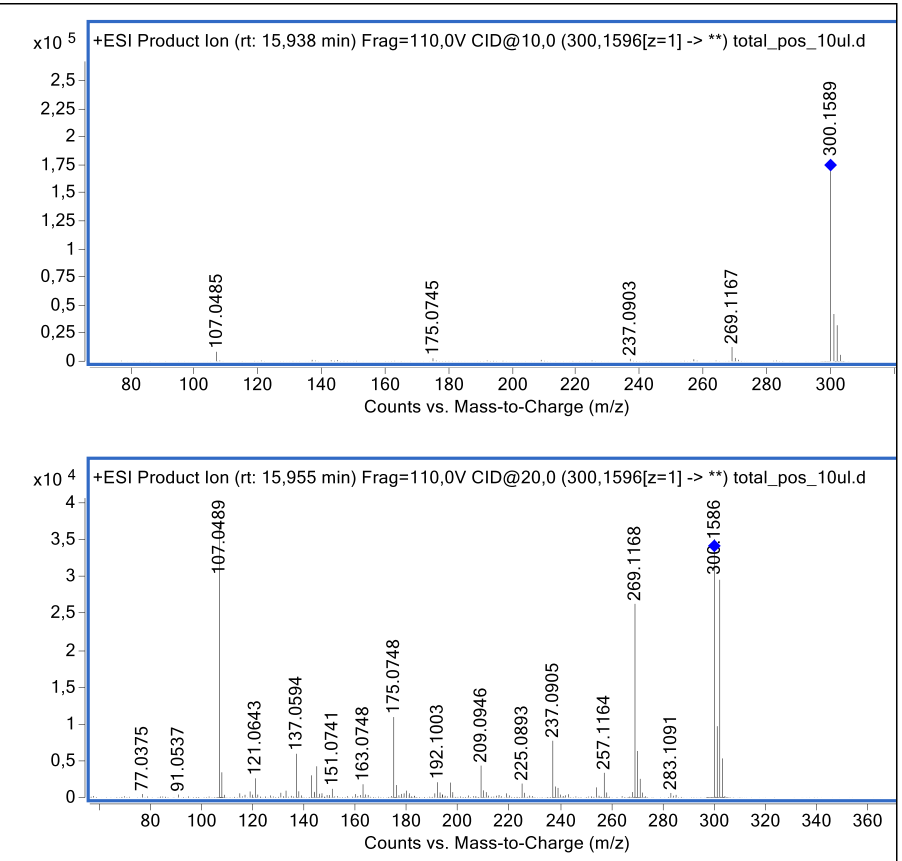

10.	16.24	Caffeic acid	$C_9H_8O_4$	179.0343	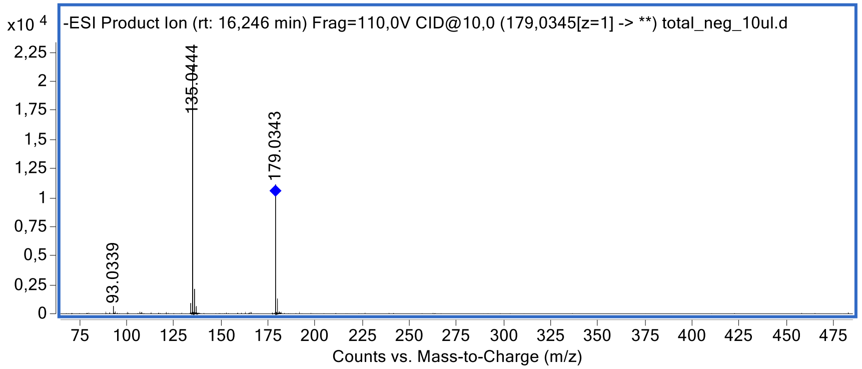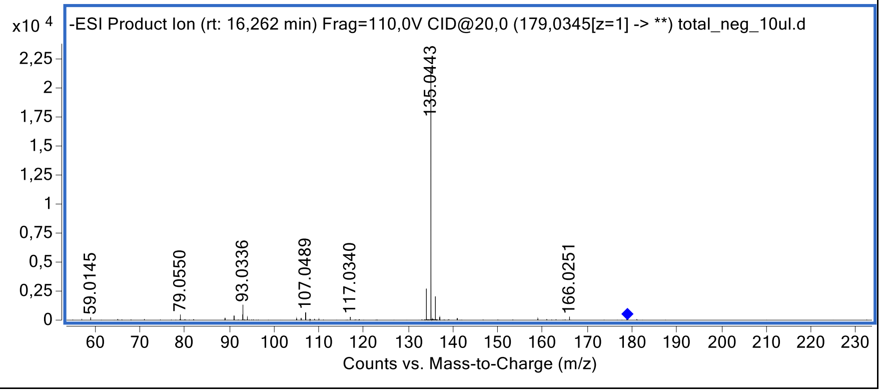

11.	16.47	2-Methylmagnocurarine	$C_{20}H_{25}NO_3$	328.1900	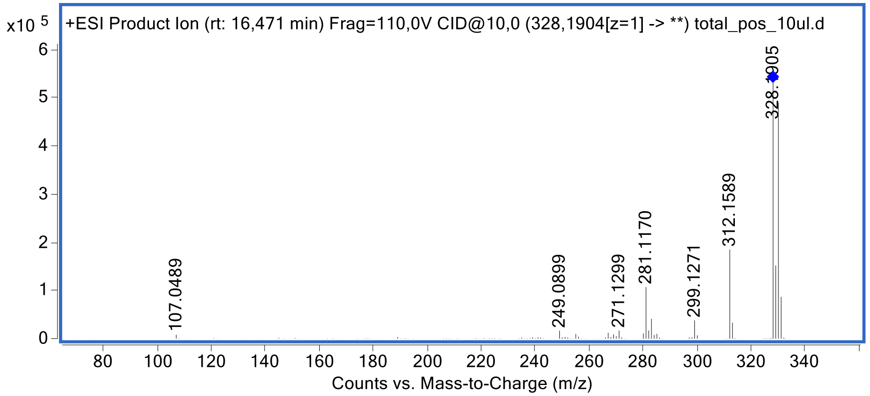<br>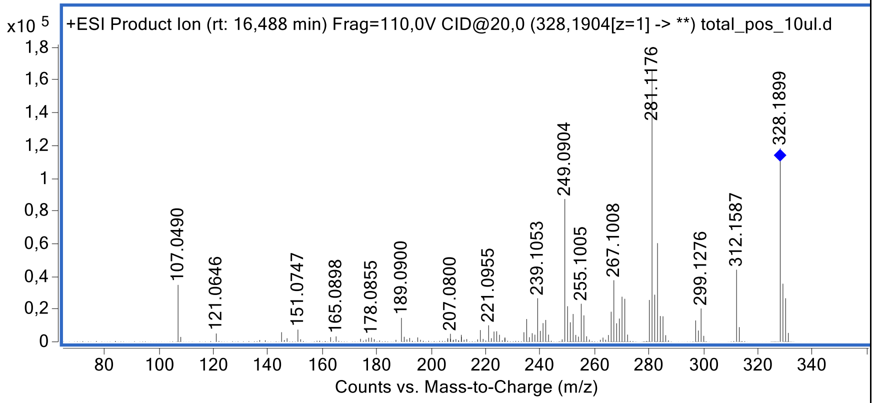

12.	16.73	Thalissopavine	$C_{20}H_{23}NO_4$	342.1700	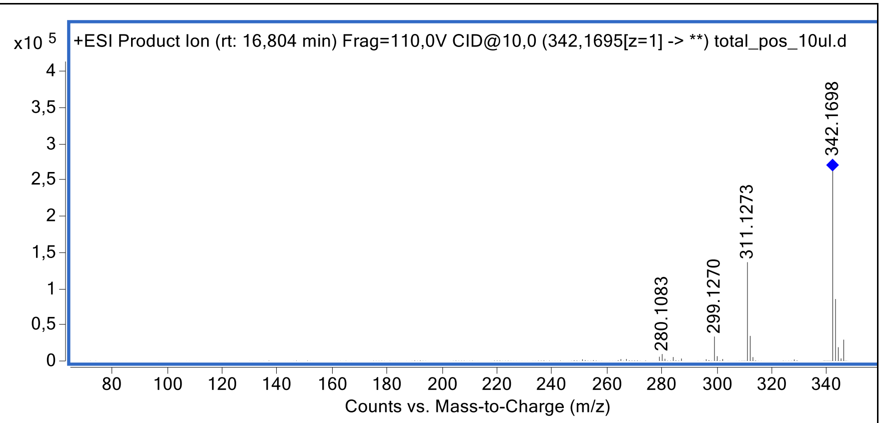 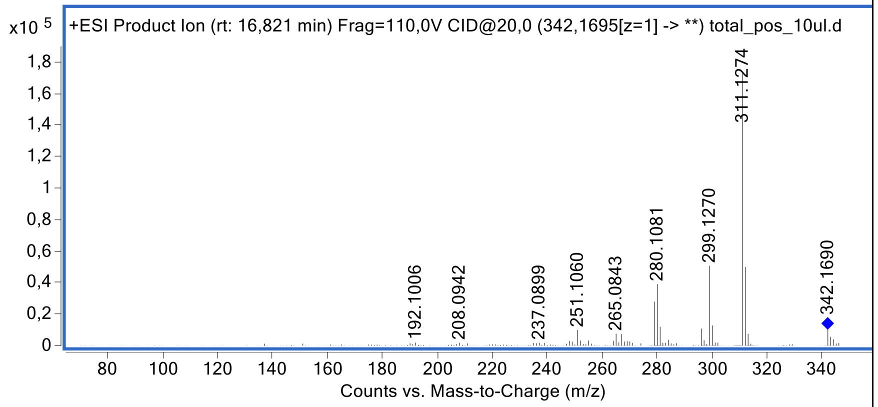

13.	16.83	Reticuline	$C_{19}H_{23}NO_4$	330.1698	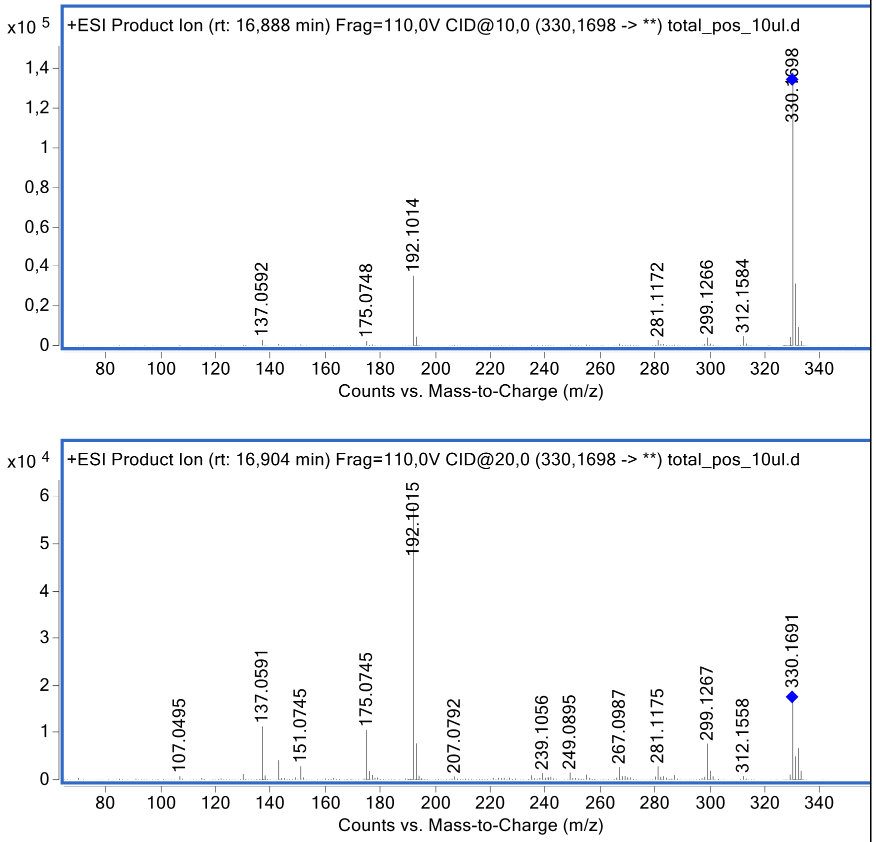

14.	17.00	Not assigned<br>(Isomer of compound 7)	n.d.	318.2057	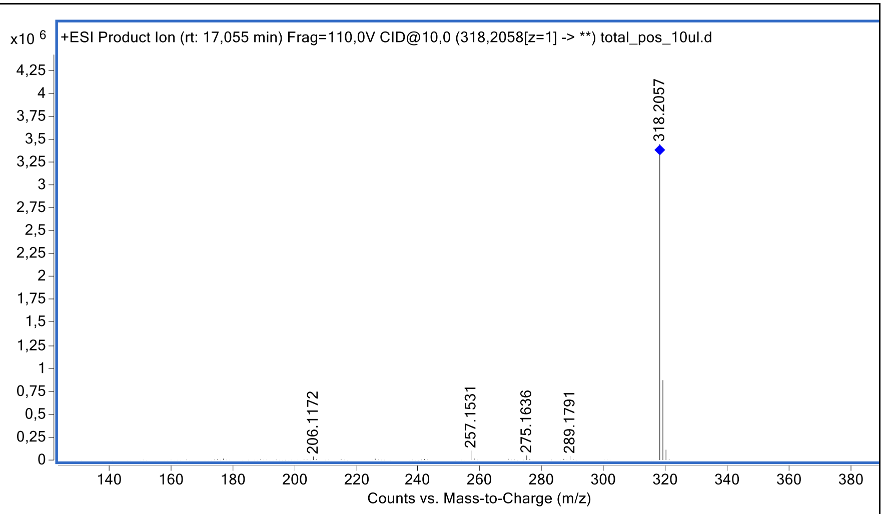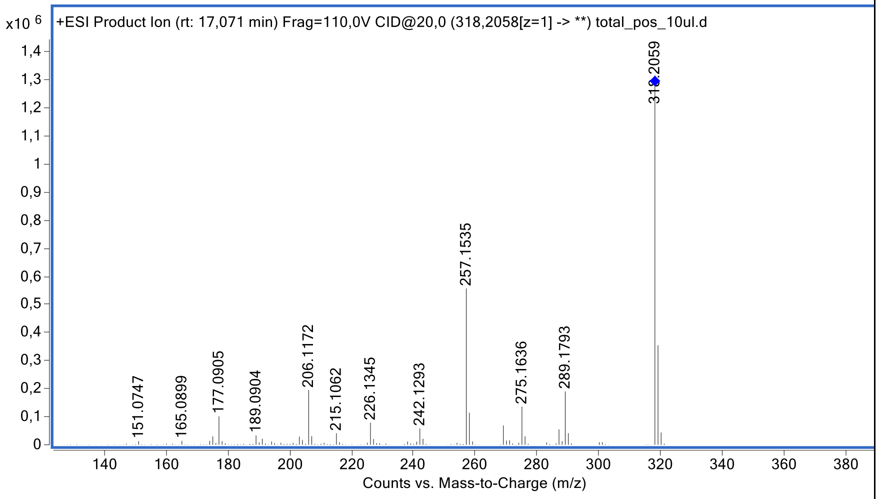

15.	17.67	Amurensine isomer 1	$C_{19}H_{19}NO_4$	326.1396	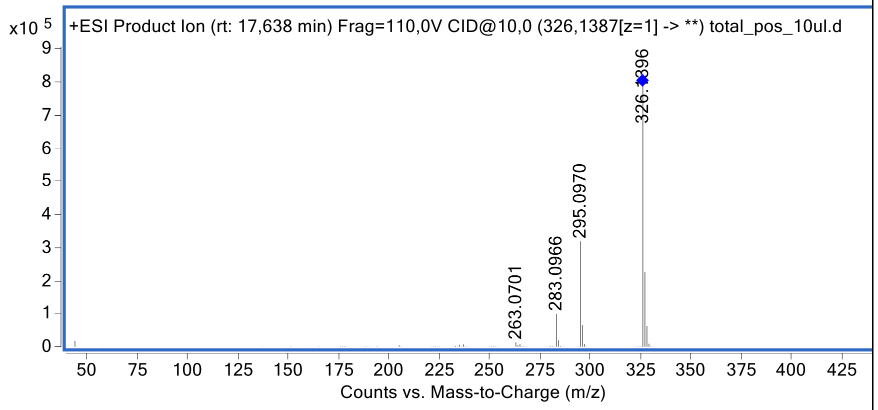 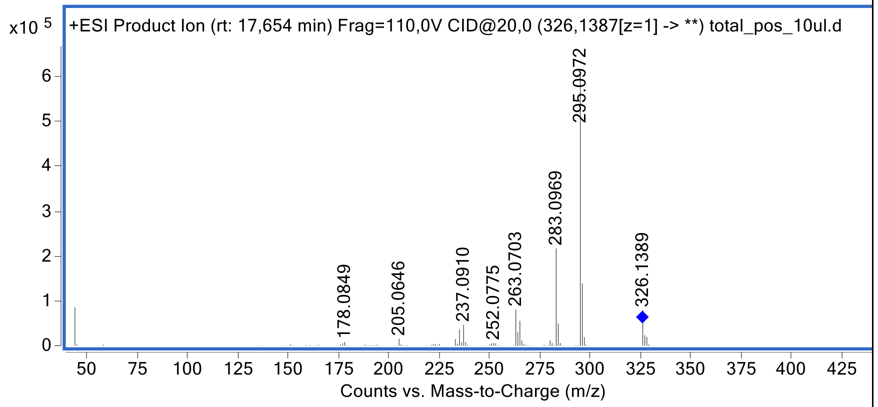
16.	17.73	Quercetin 3-O-gentiobioside	$C_{27}H_{30}O_{17}$	625.1430	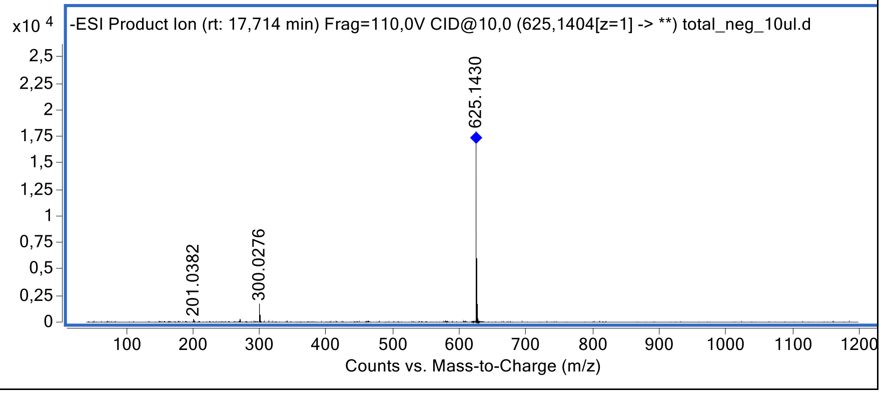

					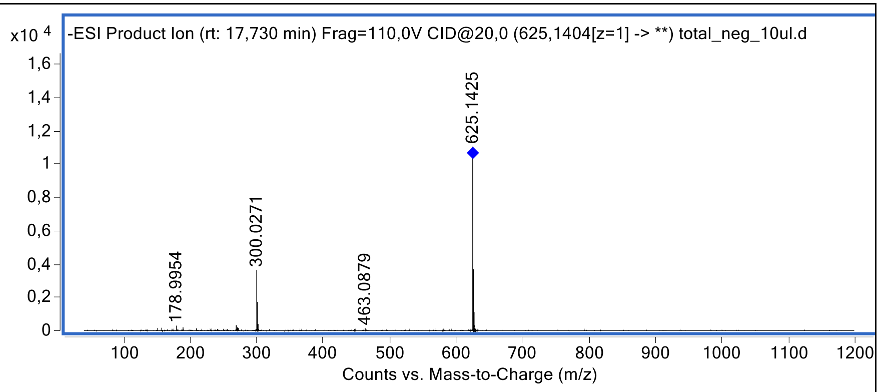
17.	18.05	Amurensine isomer 2	$C_{19}H_{19}NO_4$	326.1390	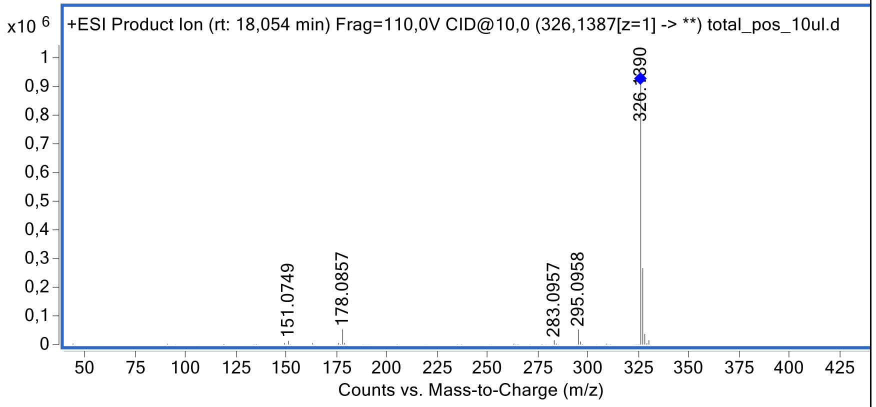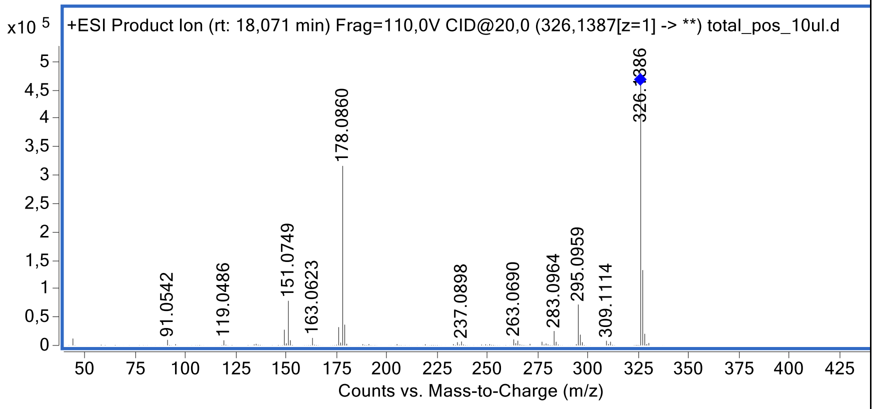

18.	18.25	Methylthalissopavine	$C_{21}H_{25}NO_4$	356.1857	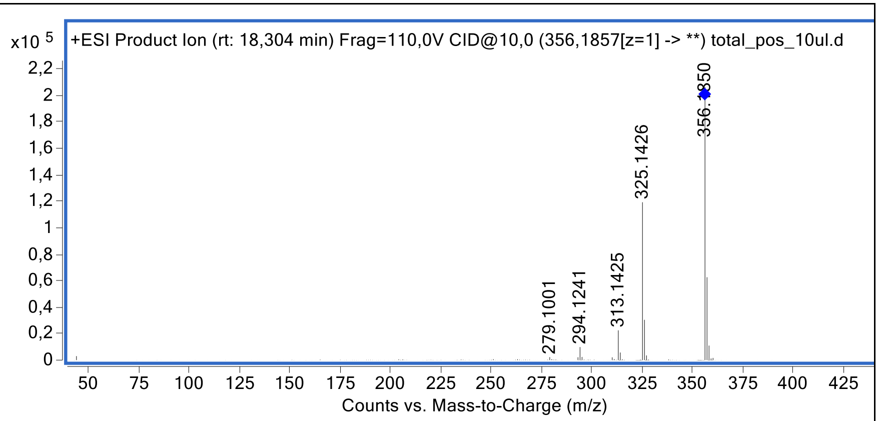 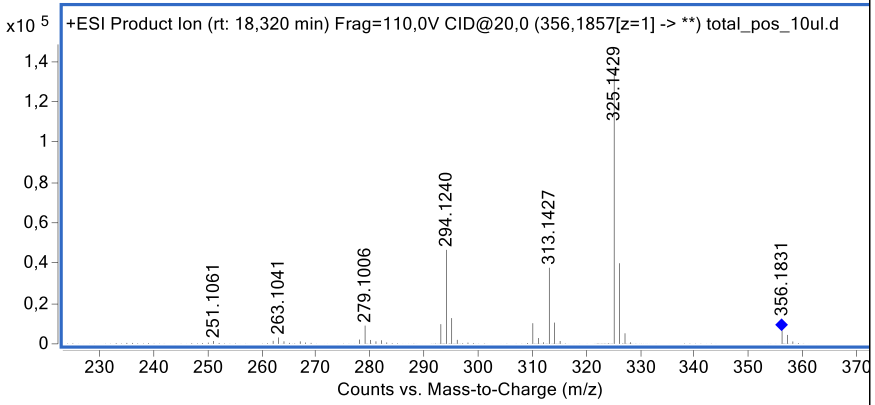

19.	18.26	Sophoraflavonoloside	$C_{27}H_{30}O_{16}$	609.1434	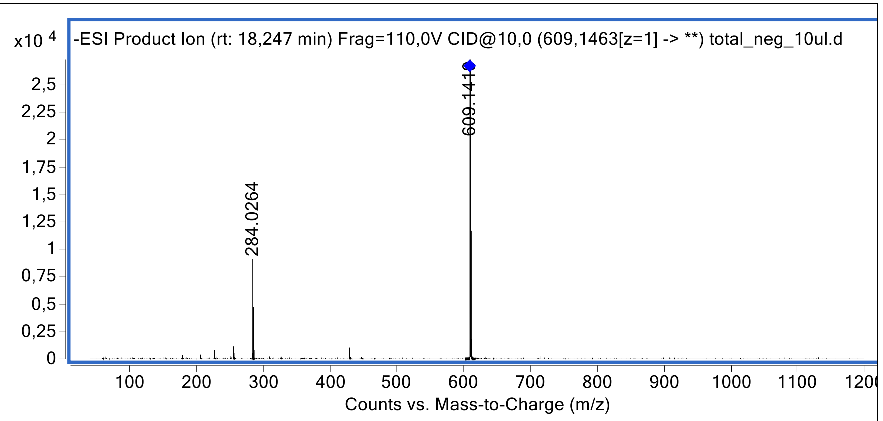 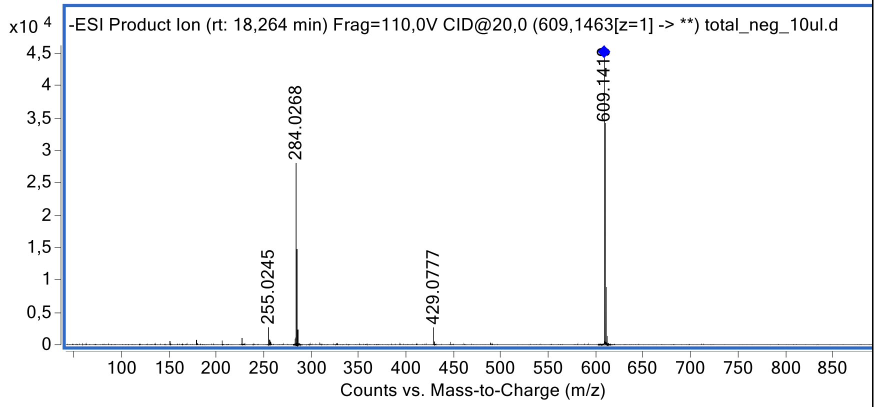

20.	18.54	Gossypitrin	$C_{21}H_{20}O_{13}$	479.0827	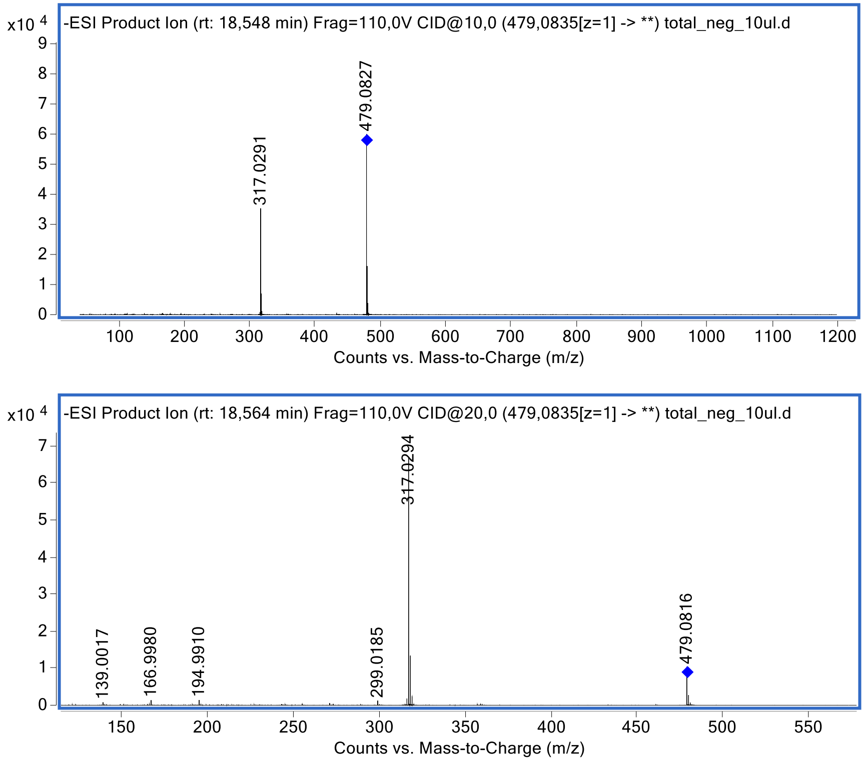

21.	18.88	ISOLATED COMPOUND	$C_{20}H_{21}NO_4$	340.1535	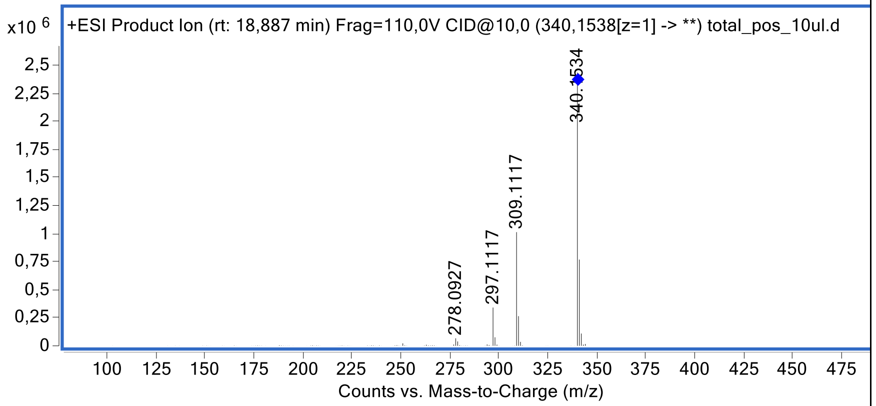 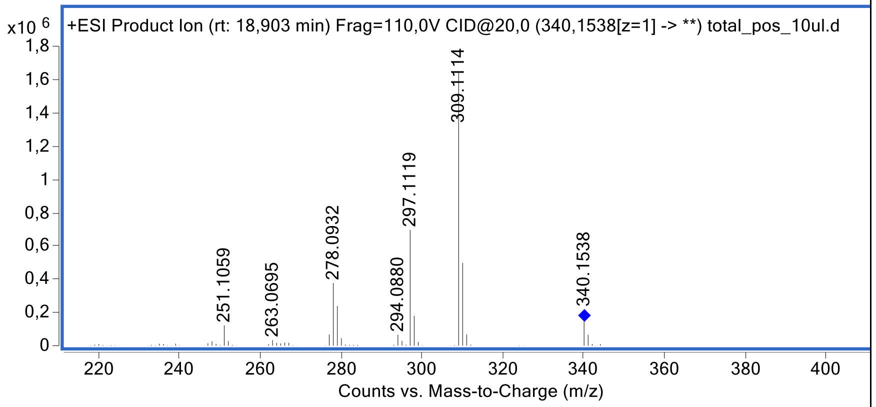

22.	19.06	Quercetin -*O*-glucoside isomer1	$C_{21}H_{20}O_{12}$	463.0882	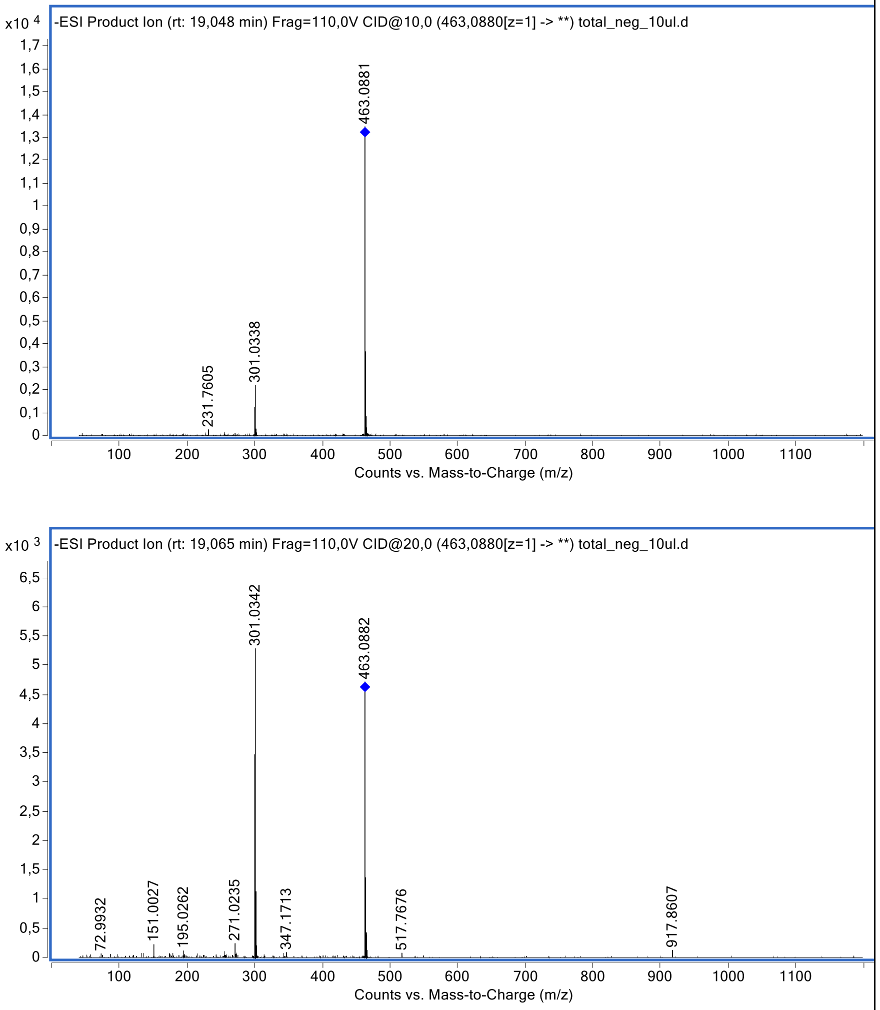

23.	19.40	Amurensinine N-oxide	$C_{20}H_{21}NO_5$	356.1490	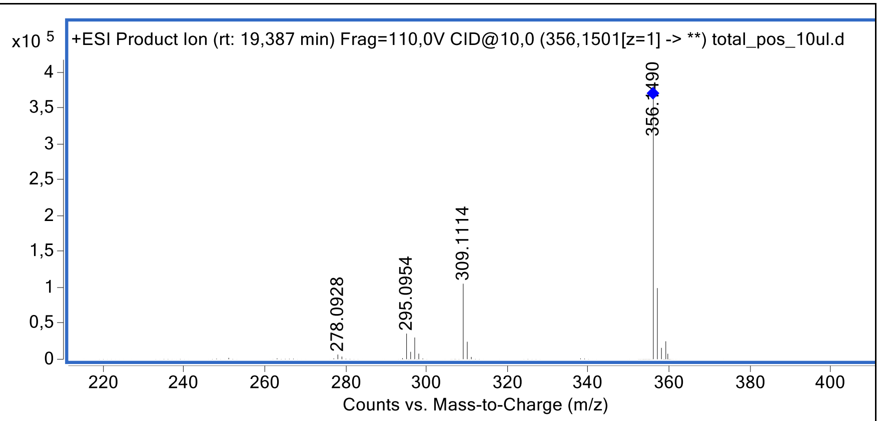 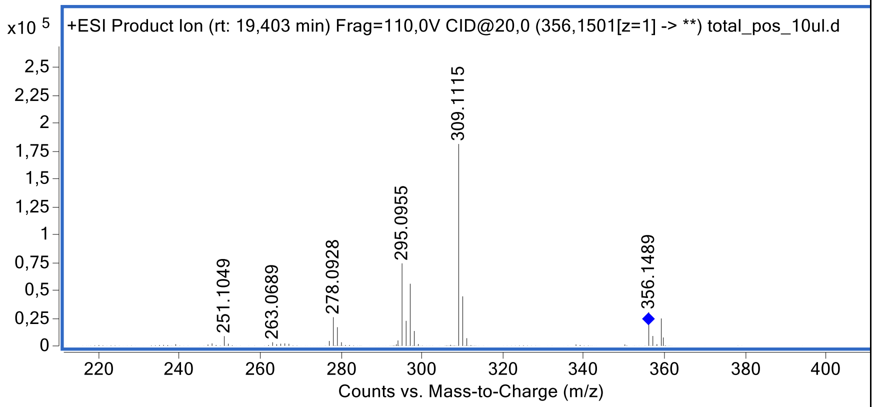

24.	19.45	Muramine	$C_{22}H_{27}NO_5$	386.1945	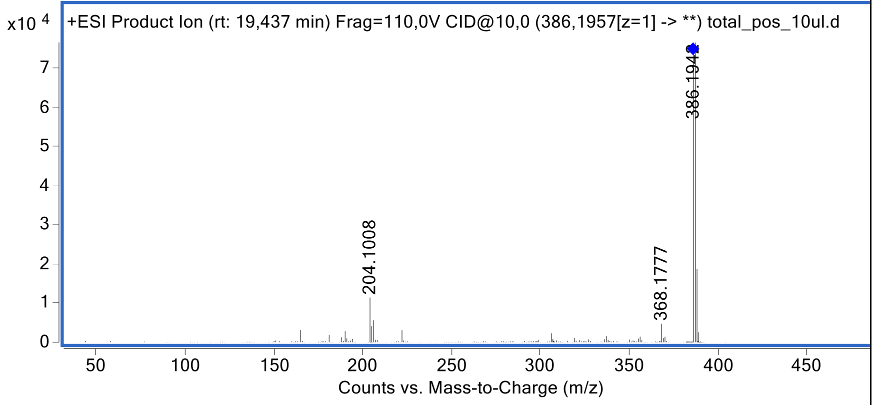 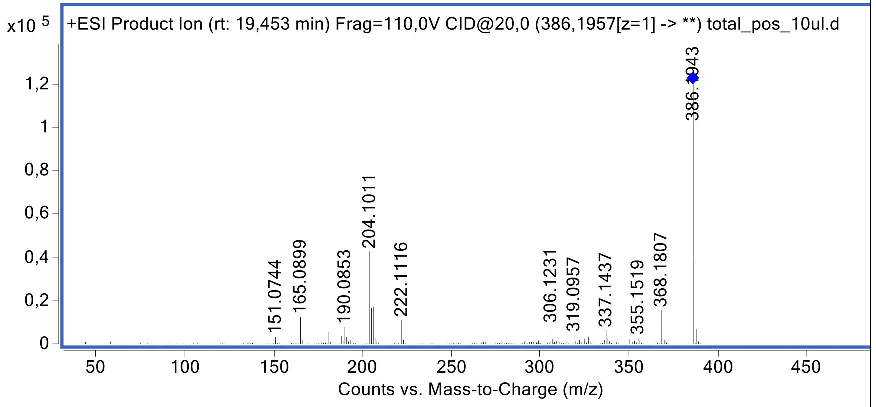

25.	19.47	Dihydrocryptopine	$C_{21}H_{25}NO_5$	372.1802	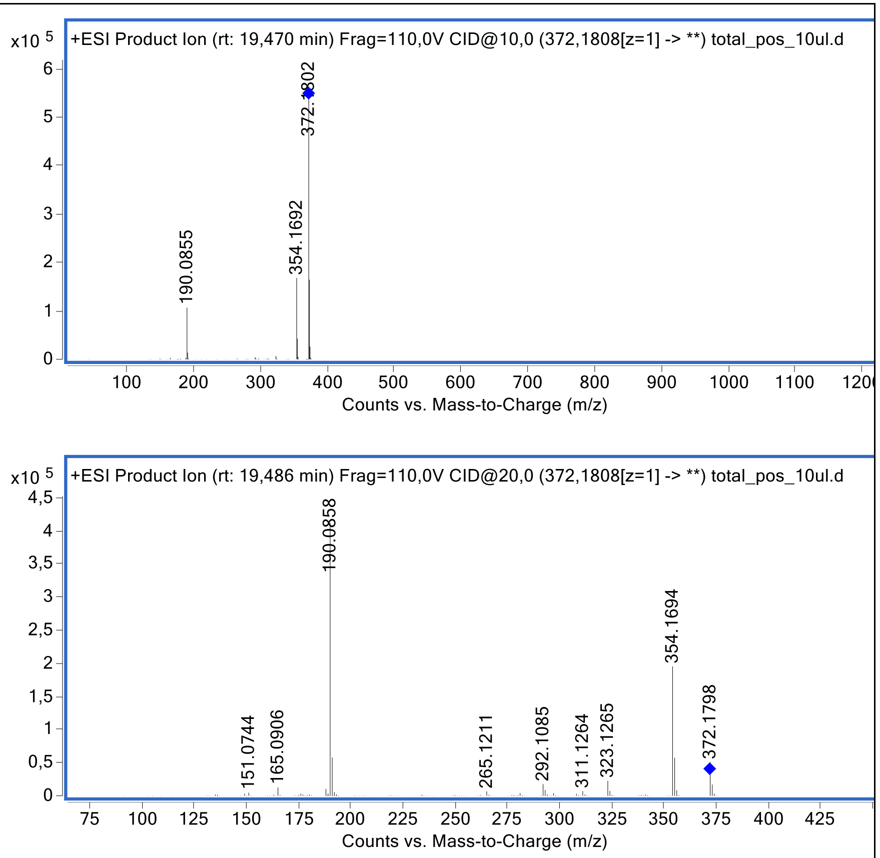
26.	19.54	Quercetin -*O*-glucoside isomer 2	$C_{21}H_{20}O_{12}$	463.0900	

					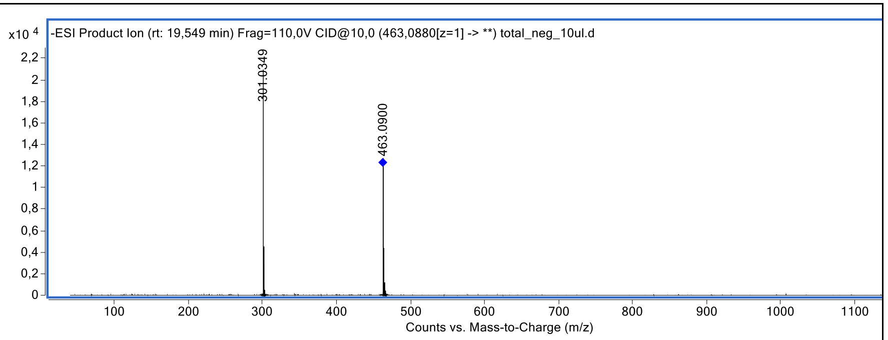 x10 4<br>-ESI Product Ion (rt: 19,549 min) Frag=110,0V CID@10,0 (463,0880[z=1] -> **) total_neg_10ul.d<br>301.0349<br>463.0900<br>Counts vs. Mass-to-Charge (m/z) 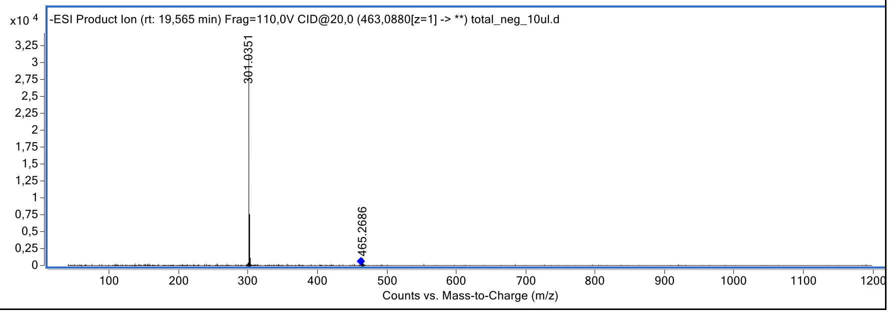 x10 4<br>-ESI Product Ion (rt: 19,565 min) Frag=110,0V CID@20,0 (463,0880[z=1] -> **) total_neg_10ul.d<br>301.0351<br>465.2686<br>Counts vs. Mass-to-Charge (m/z)

27.	19.73	α-Allocryptopine	$C_{21}H_{23}NO_5$	370.1646	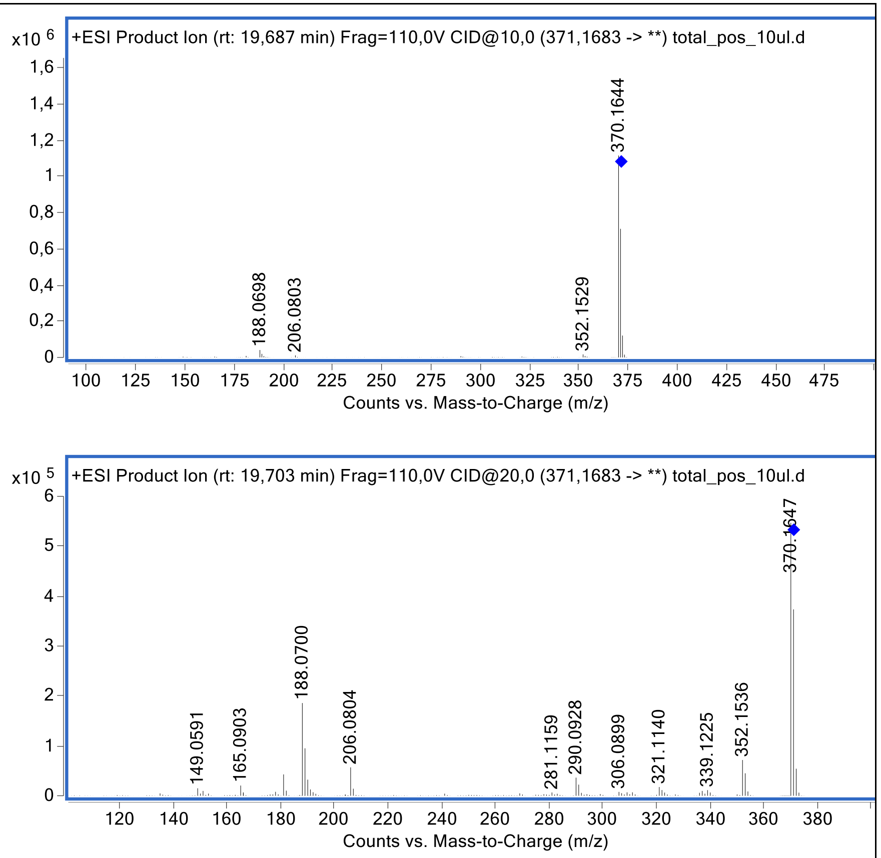

28.	19.80	Reframidine	$C_{19}H_{17}NO_4$	324.1219	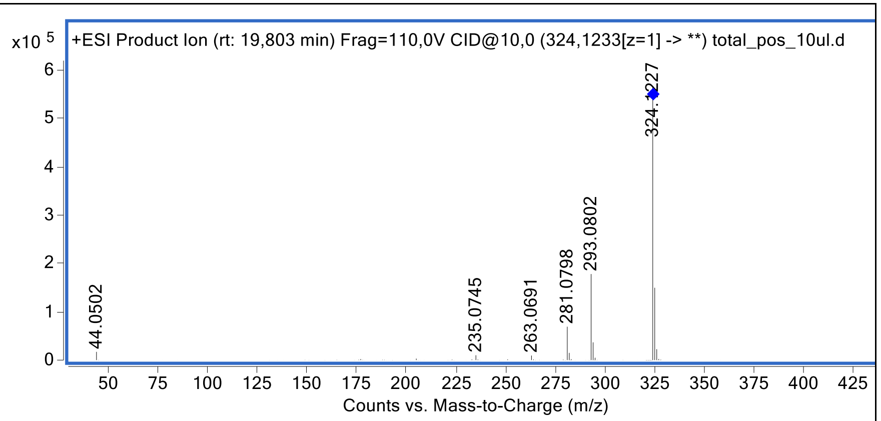<br>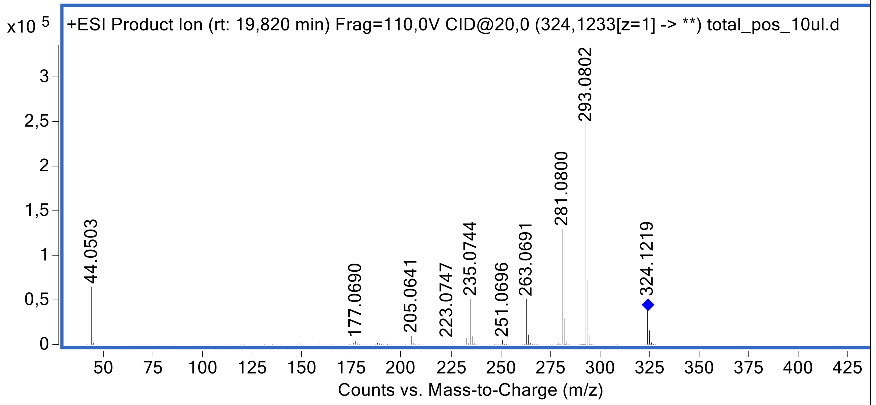

29.	20.21	Cryptopine	$C_{21}H_{23}NO_5$	370.1647	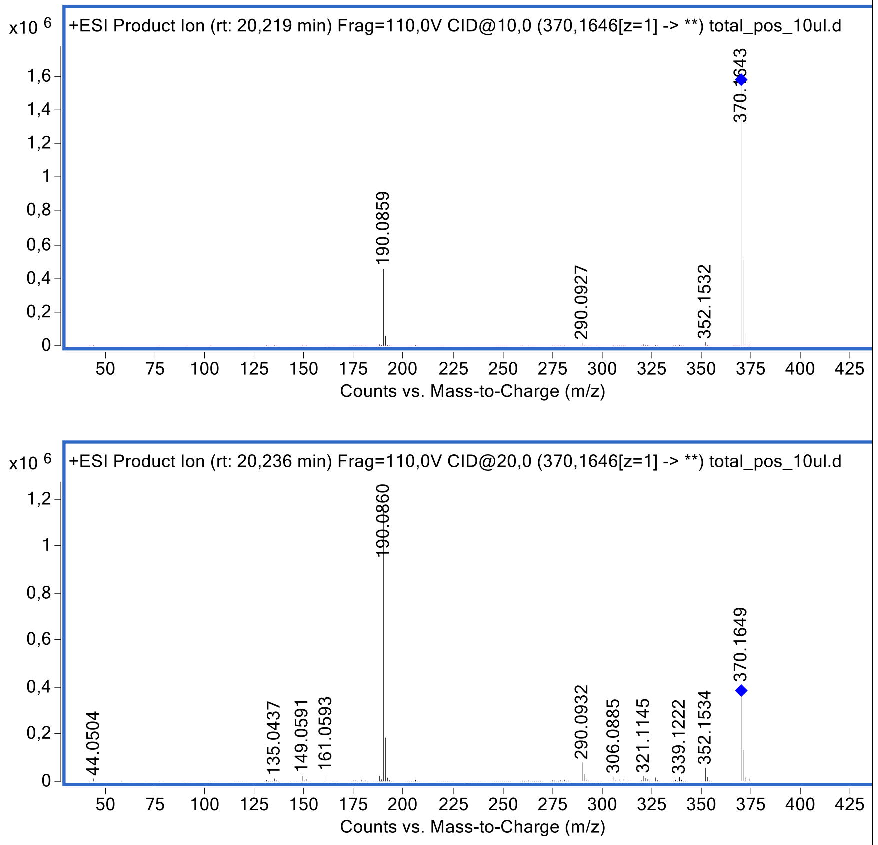

30.	20.68	N-Methylcanadine	$C_{21}H_{24}NO_4^+$	354.1699	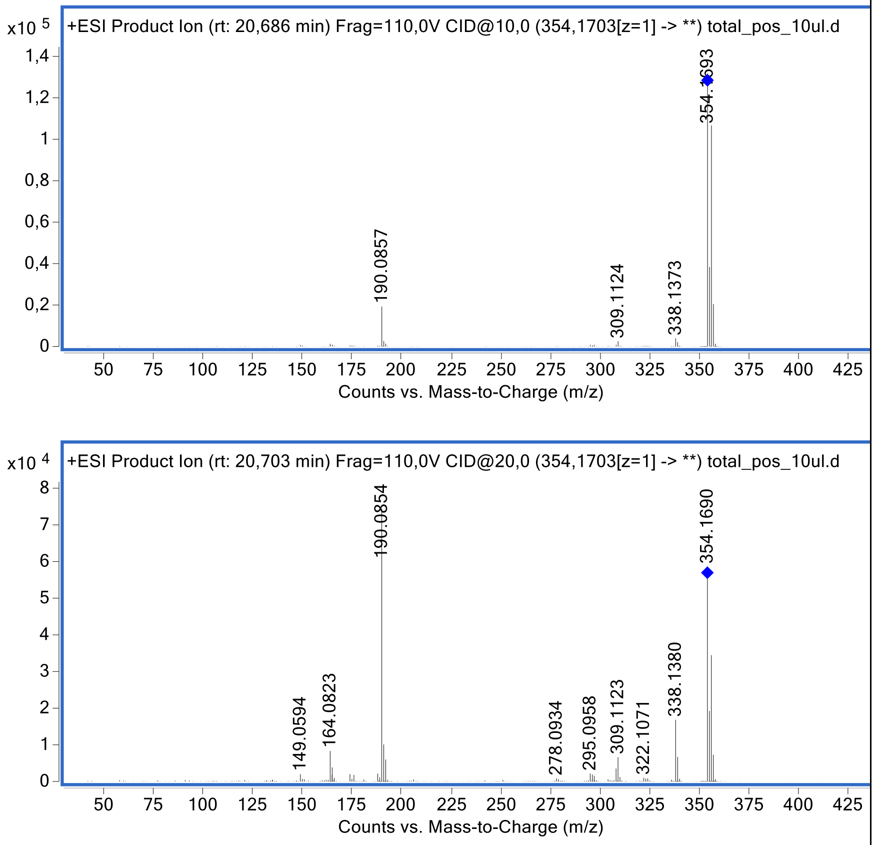

31.	20.81	Alborine	$C_{22}H_{22}NO_6^+$	396.1436	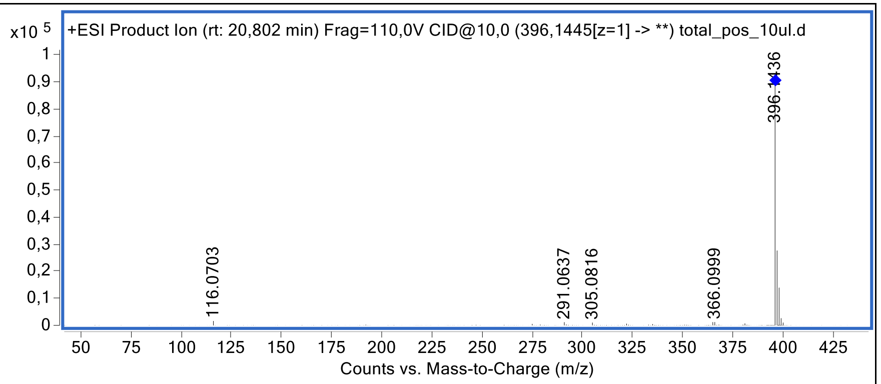 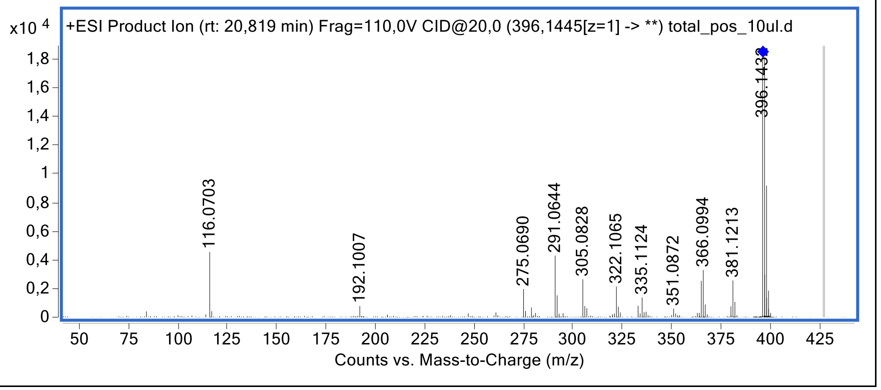

32.	20.98	Berberine	$C_{20}H_{18}NO_4^+$	336.1233	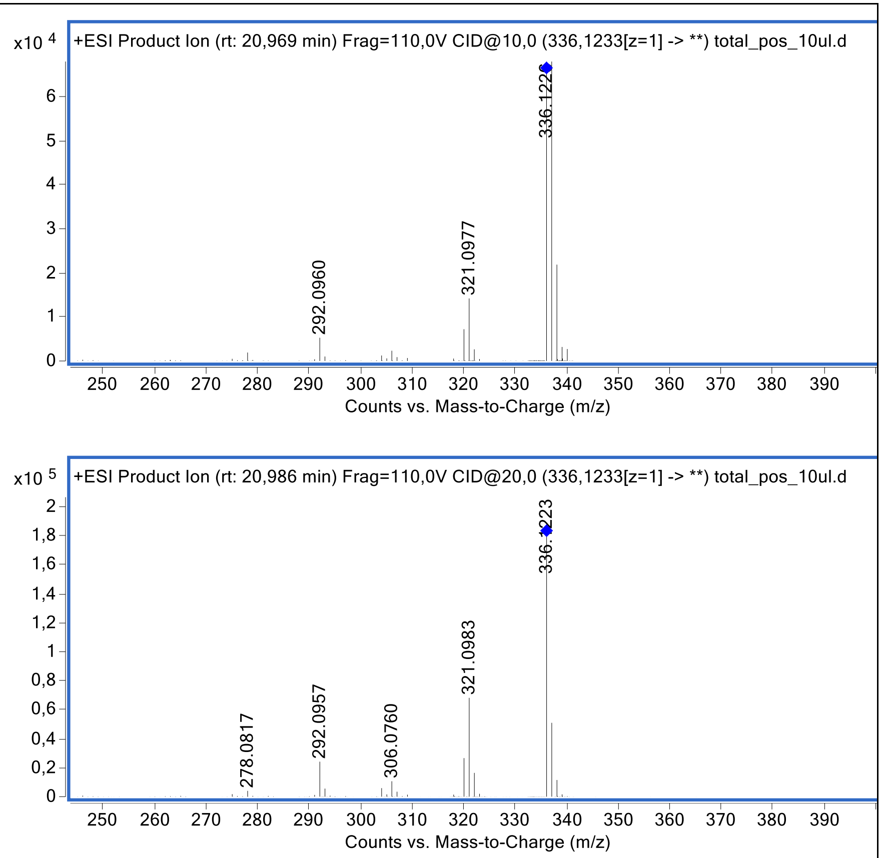

33.	21.60	Luteolin	$C_{15}H_{10}O_6$	285.0406	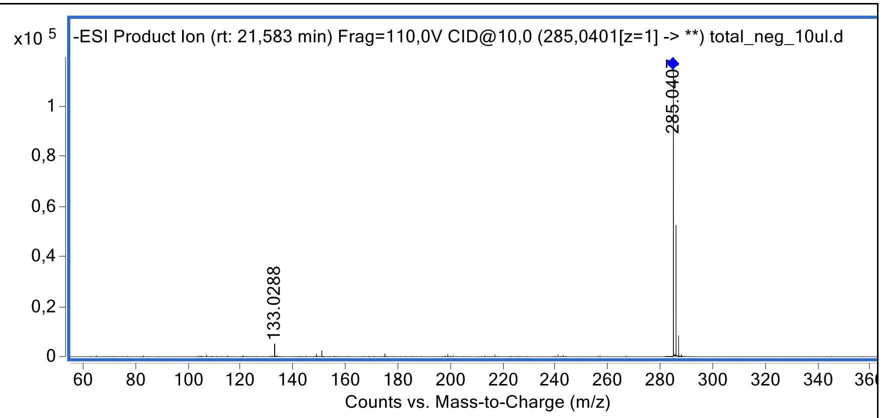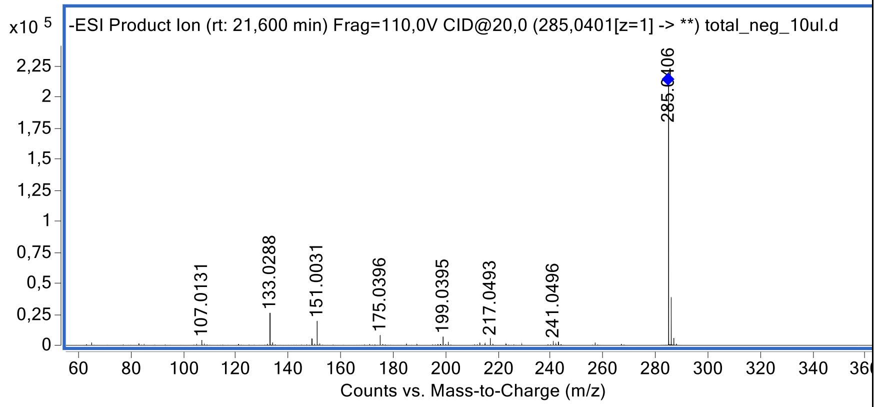

34.	21.33	Not assigned	n.d.	274.2734	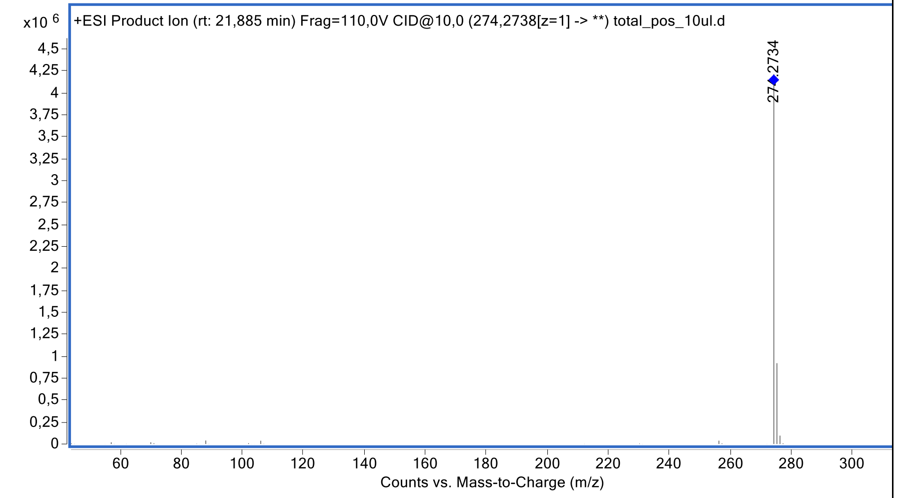 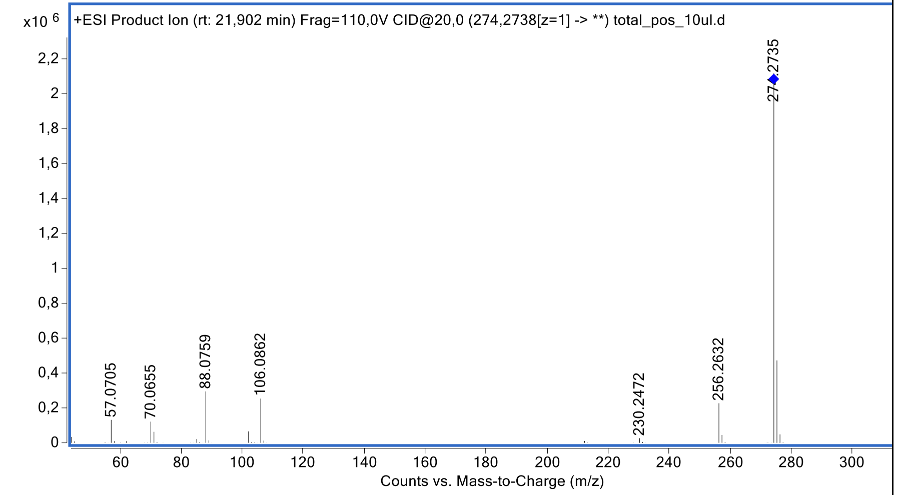

molecules

MDPI

Table S2. The partition coefficient values obtained for the investigated biphasic solvent systems

n*-hexane/*n*-BuOH/EtOH/H_2O 3:12:6:15 *v/v/v/v						
	2u		2L			
R,time	Area	R,time	Area		Coefficient (K_D)	
12.643	1635404	12.630	8905640		0.183636886	
18.854	4568396	15.776	2471720		1.848265985	
20.191	2667164	18.867	9629593		0.276975777	
21.146	1839443	20.238	6923368		0.265686152	
22.090	9378099	21.236	3389609		2.766719996	
22.685	8092842	22.098	11070803		0.731007678	
25.461	6645251	22.689	8901184		0.746558098	
27.494	11692509	24.663	647945		18.04552701	
		25.638	5266616			
		27.761	2591886			
n*-hexane/*n*-BuOH/EtOH/H_2O, 1:14:6:15 *v/v/v/v						
	3u		3L			
R,time	Area	R,time	Area		Coefficient (K_D)	
18.860	1421584	18.888	6393399		0.222351835	
20.204	1242379	20.266	4600119		0.270075405	
21.151	863181	21.271	2290392		0.376870422	
22.107	4563906	22.145	8205768		0.556182675	
22.718	3431394	22.729	7065028		0.485687247	
24.632	154327					
25.462	2762873	25.644	4593445		0.601481677	
27.464	875734					
30.284	717909	30.301	1864411		0.38505941	
**MtBE/*n*-BuOH/ACN/HCl 2:2:1:5 *v/v/v/v***						
	4u		4L			
R,time	Area	R,time	Area		Coefficient (K_D)	
18.540	504769	18.842	1899875		0.265685374	
20.178	882802	20.227	2325883		0.379555635	
21.154	769826	21.145	907241		0.848535284	
22.140	4424959	21.831	3345673		1.322591598	
22.762	3191055	22.939	2665335		1.197243498	
25.466	3201368	25.645	1232834		2.596755119	
27.499	984422	27.736	82211		11.97433433	
n*-hexane/EtOAc/EtOH/H_2O 5:3:4:4 *v/v/v/v						
	6u		6L			
R,time	Area	R,time	Area		Coefficient (K_D)	

13.062	52862					
14.303	55721	15.808	12641864		0.004407657	
		20.249	3970613		0	
21.827	105231	21.171	2942654		0.035760575	
		22.128	7568201		0	
23.057	69815	22.857	6595090		0.010585906	
30.393	119107	24.708	1140662		0.104419188	
33.625	25431	25.469	4406602		0.005771113	
36.094	92393	27.562	4061315		0.022749528	
45.186	70345					
47.290	26186					
50.261	861332					

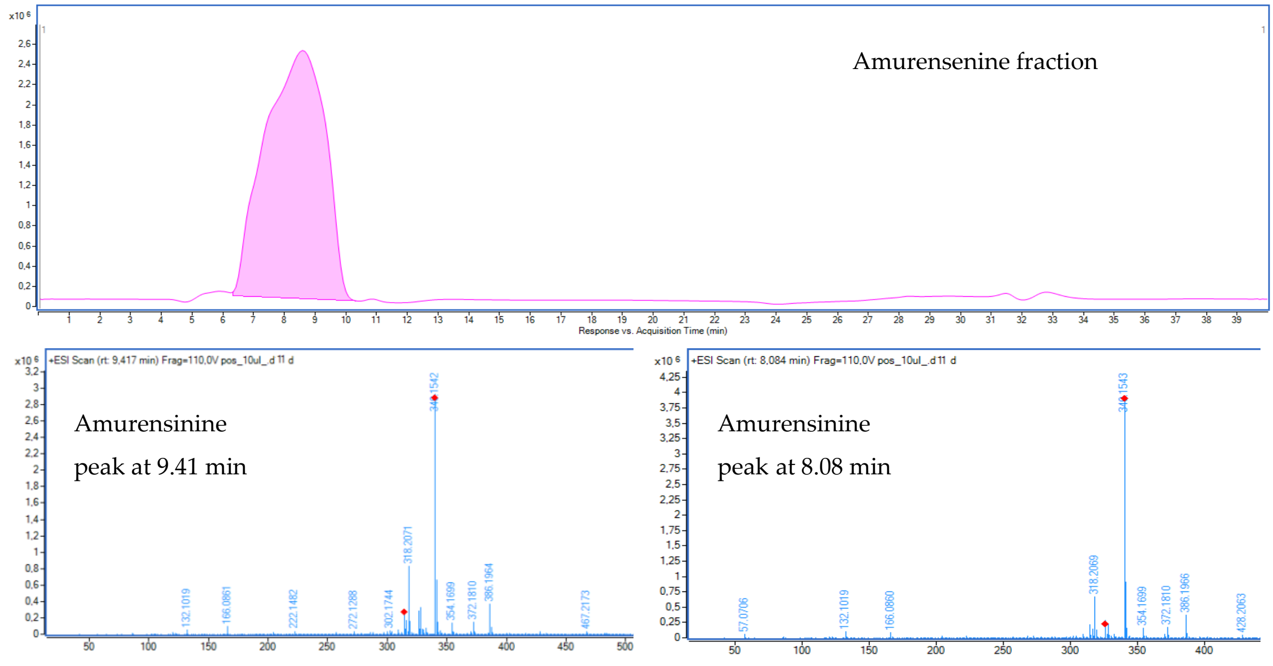

Figure S1. The TIC chromatogram of amurensinine (fraction 3) in the HPLC-MS analysis with the MS spectra for two selected retention times (at 9.41 min and 8.08 min), proving its purity. The purity result obtained from the program was 96.2%.

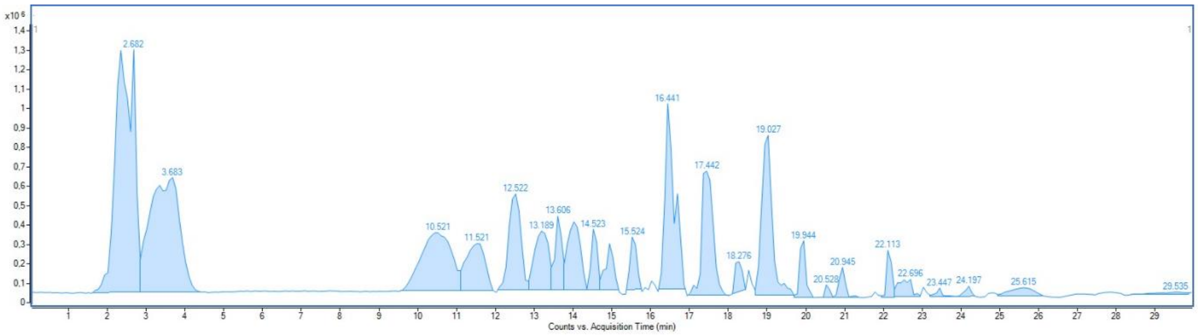

Figure S2. The HPLC-MS fingerprint of the fraction 2 from CPC separation in the negative ion mode used for the acetylcholinesterase inhibition assay

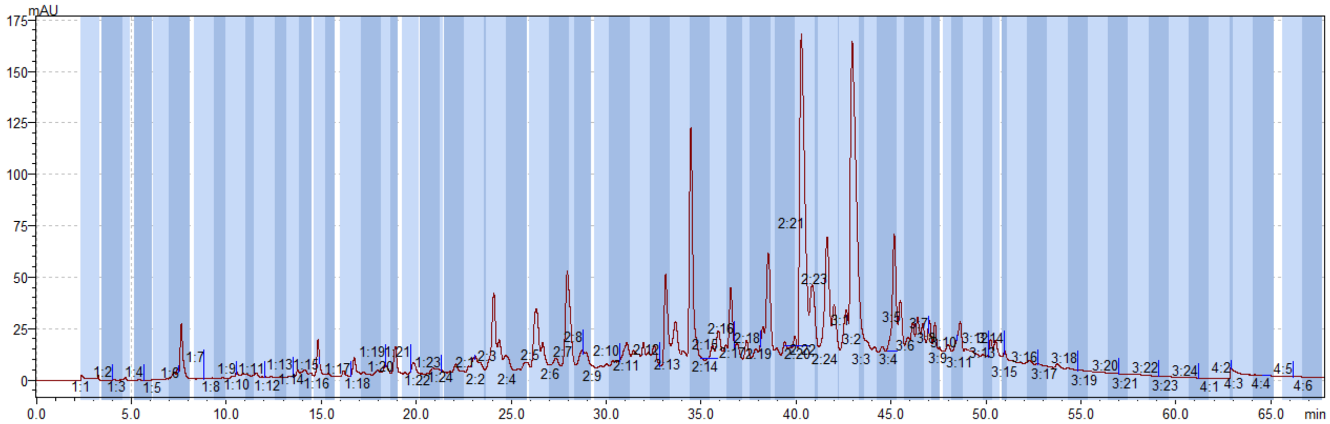

Figure S3. The semi-preparative HPLC chromatogram of fraction 3 from CPC separation, recorded at 290 nm with amurensinine with the retention time of 40 min (fraction 2:21).

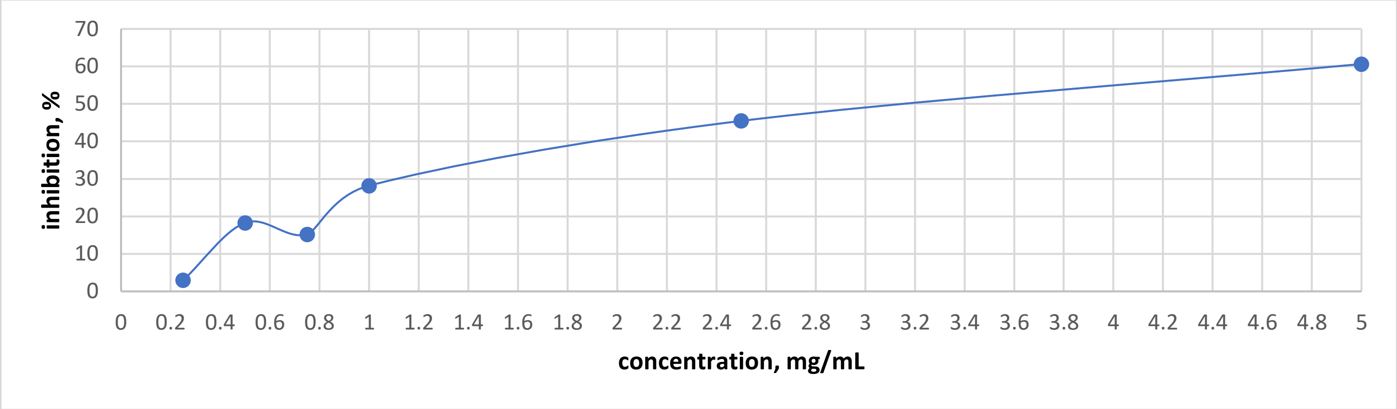

Figure S4. Concentration-dependent inhibition of AChE by amurensinine-isolated from fraction 3 in FAIA. The calculated IC_{50} value is 3.08 mg/mL

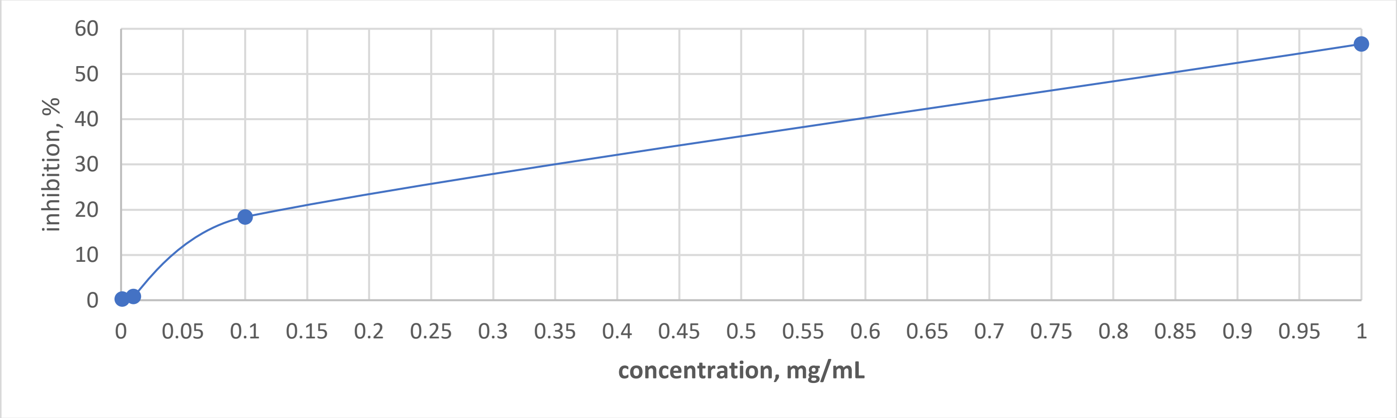

Figure S5. Concentration-dependent inhibition of AChE by berberine standard (positive control) in FAIA. The calculated IC_{50} value is 0.84 mg/mL

TableS3. Assigned ^{1}H and ^{13}C NMR chemical shifts of amurensinine recorded in DMSO-d_6 at 25 °C.

Atom	Nucleus	δ / ppm
C1	13C	107.633
C1′	13C	135.820
C10	13C	114.902
C10′	13C	135.440
C11	13C	37.962
C12	13C	61.754
C2′	13C	100.762
C2/3	13C	145.857
C4	13C	106.351
C4′	13C	131.679
C5	13C	44.800
C6	13C	59.743
C7	13C	112.299
C7′	13C	127.000
C8	13C	147.758
C9	13C	146.694
CMe8	13C	55.977
CMe9	13C	56.048
CNMe	13C	45.330
H1	1H	6.855
H10	1H	6.547
H11a	1H	2.739
H11b	1H	3.338
H12	1H	3.866
H2′a	1H	5.870
H2′b	1H	5.941
H4	1H	6.818
H5	1H	3.745
H6a	1H	2.704
H6b	1H	3.336
H7	1H	6.762
HMe8	1H	3.635
HMe9	1H	3.714
HNMe	1H	2.333

Note. The notation C2/3 denotes unresolved or jointly assigned carbon resonances corresponding to positions C2 and C3.

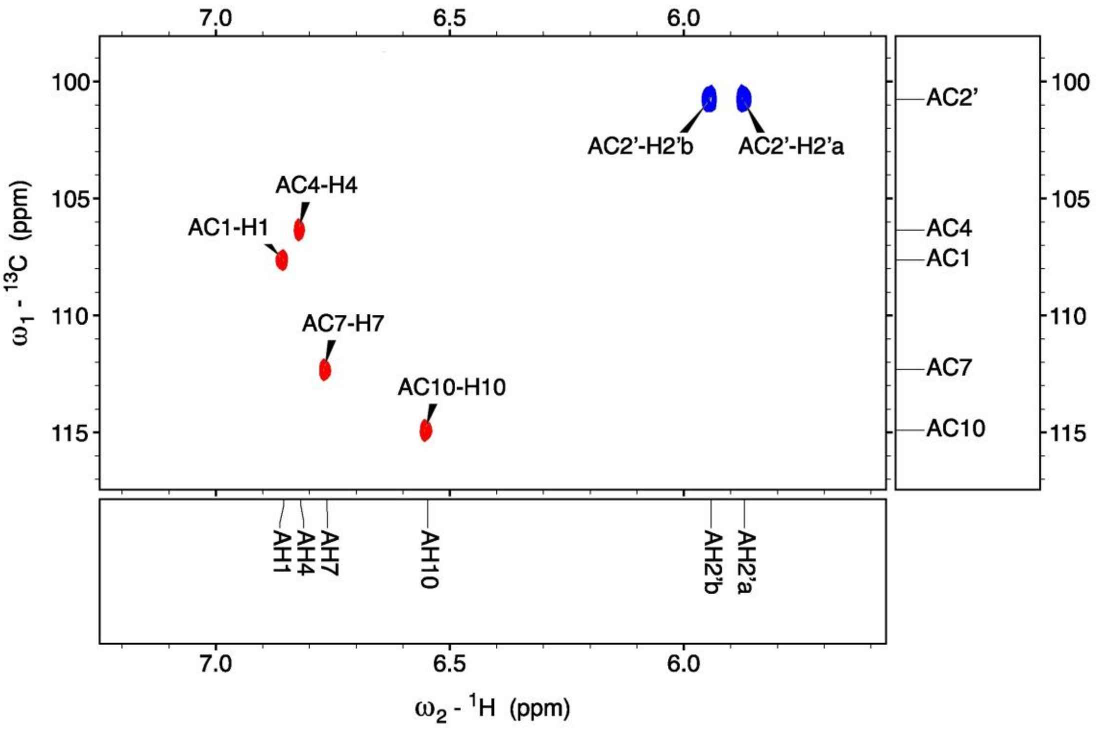

Figure S6. The diagnostic region of the edited ^{1}H-^{13}C HSQC spectrum. Red colour corresponds to the positive-phase cross-peaks, indicating methyl (CH_3) and methine (CH) groups. The blue colour represents negative-phase correlations, indicating the presence of methylene (CH_2) groups.

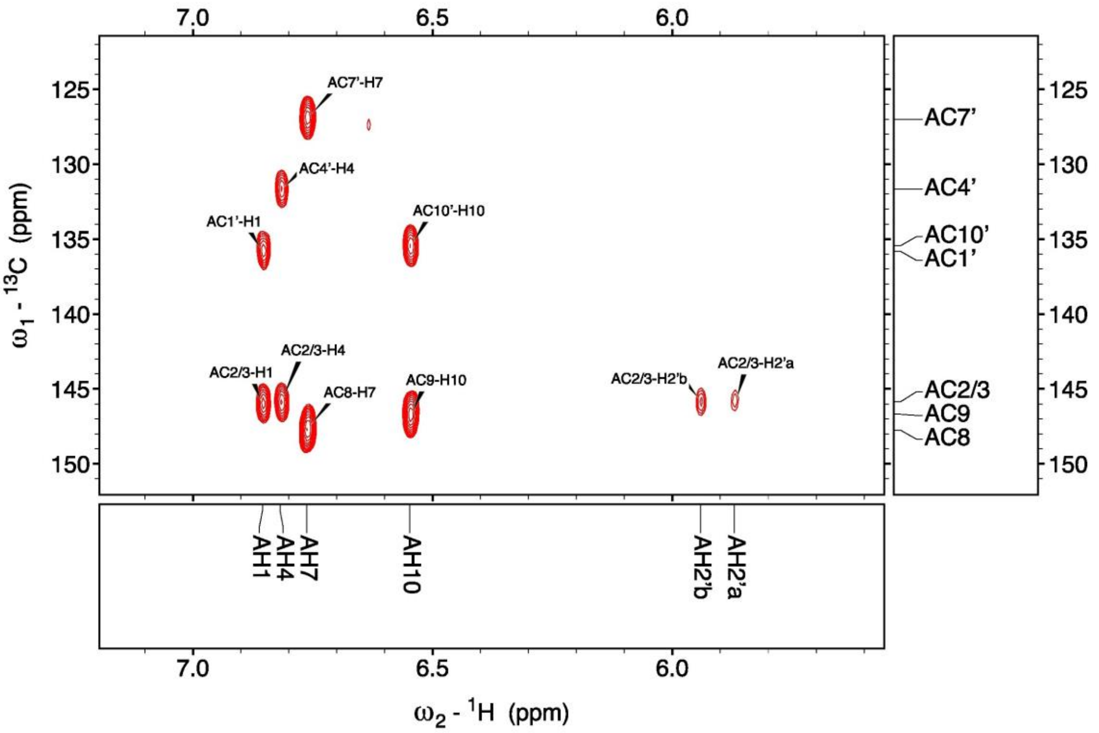

Figure S7. The diagnostic region of the edited ^{1}H-^{13}C HMBC spectrum.

Table S4. The NMR data obtained for the purified amurensinine

1	1H NMR spectrum of amurensinine. The spectrum was acquired with 256 scans.
	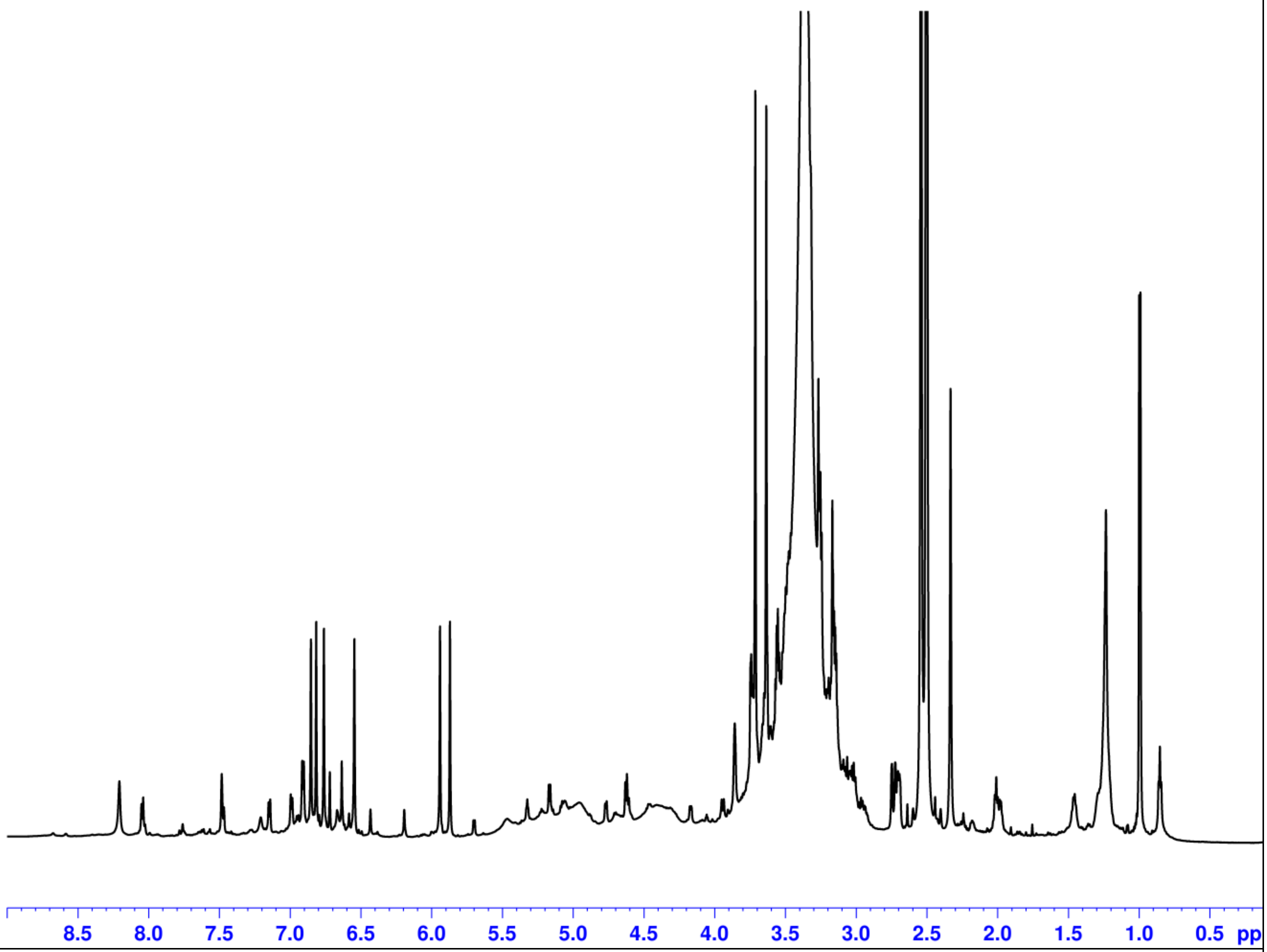
2	13C Spectrum of amurensinine. The spectrum was acquired with 2532 scans.

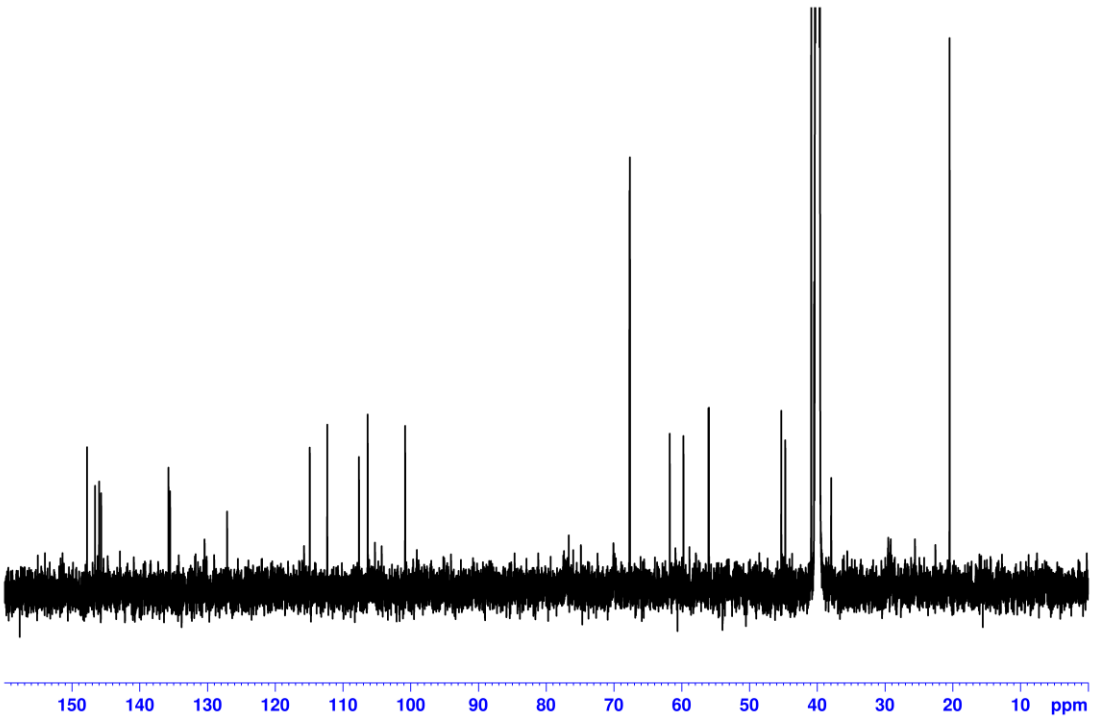

3 ^{1}H–^{1}H DQF-COSY spectrum of amurensinine recorded in DMSO-d$_6$ at 25 °C on a 700 MHz NMR spectrometer equipped with a QCI-CryoProbe. The spectrum was acquired with 112 scans and 256 increments in the F1 dimension. The spectral width was 7716 Hz in the ^{1}H dimension. The acquired data matrix was 2048 × 256 points and the spectrum was processed using a final matrix size of 2K × 1K.

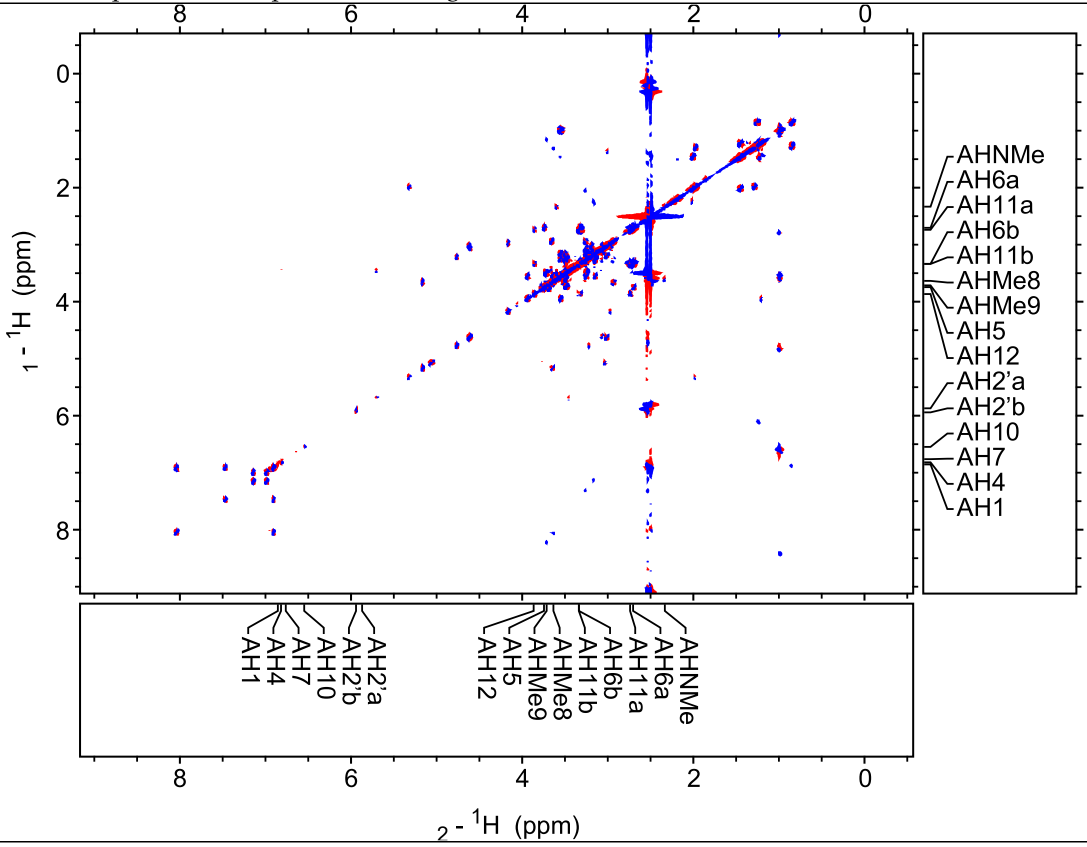

4	$^{1}H–^{1}H$ NOESY spectrum of amurensinine recorded in DMSO-d_6 at 25 °C on a 700 MHz NMR spectrometer equipped with a QCI-CryoProbe. The spectrum was acquired with 64 scans and 256 increments in the F1 dimension. The spectral width was 7716 Hz in the ^{1}H dimension. The acquired data matrix was 2048 × 256 points and the spectrum was processed using a final matrix size of 2K × 1K.
	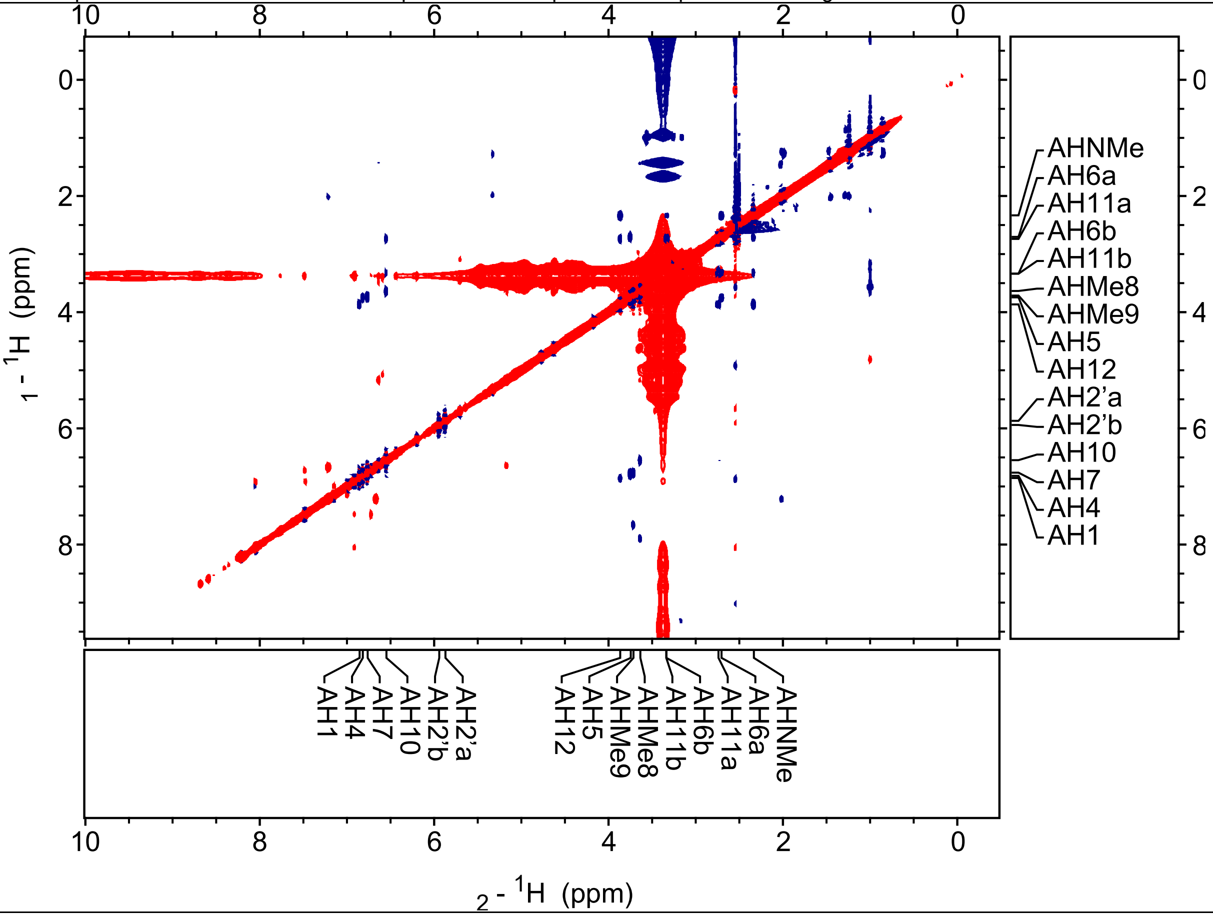
5 .	$^{1}H–^{13}C$ HSQC spectrum of amurensinine recorded in DMSO-d_6 at 25 °C on a 700 MHz NMR spectrometer equipped with a QCI-CryoProbe. The spectrum was acquired with 16 scans and 256 increments in the F1 dimension. The spectral width was 7716 Hz in the ^{1}H dimension and 29177 Hz in the ^{13}C dimension. The acquired data matrix was 2048 × 256 points and the spectrum was processed using a final matrix size of 2K × 1K.
	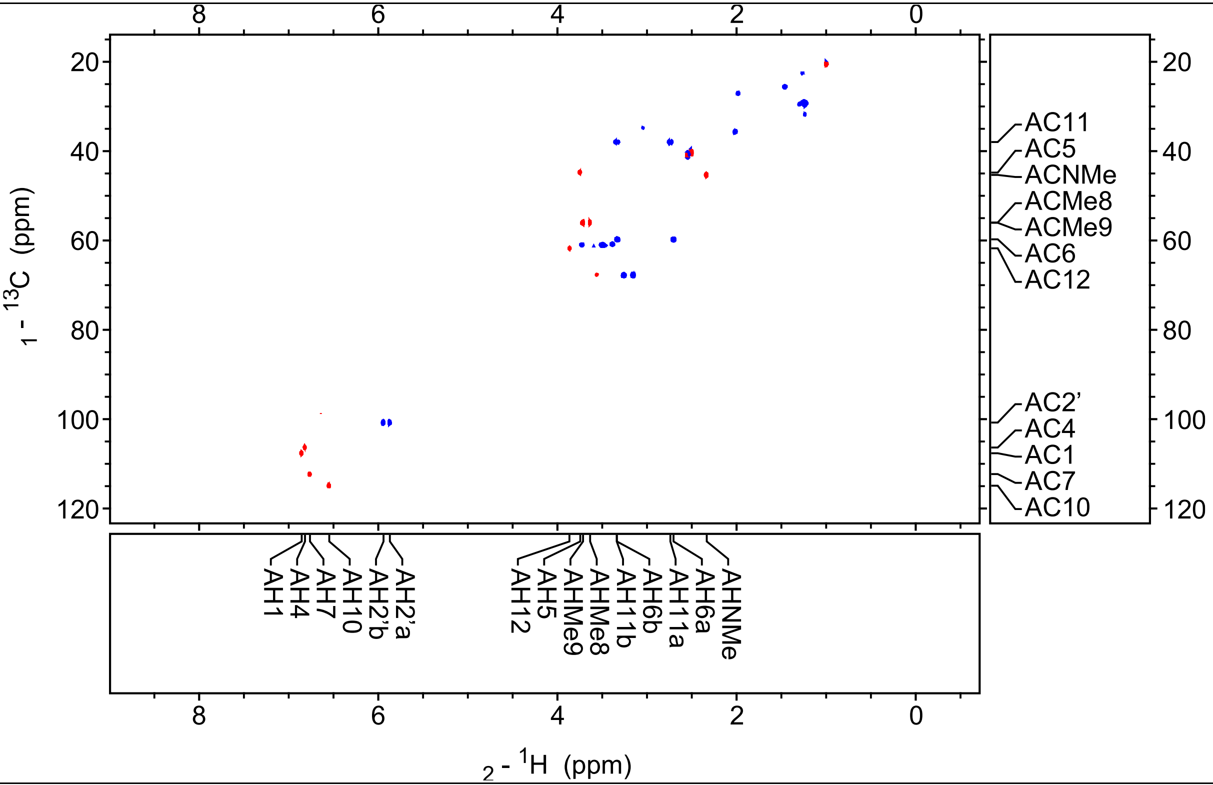

6	^{1}H–^{13}C HMBC spectrum of amurensinine recorded in DMSO-d_6 at 25 °C on a 700 MHz NMR spectrometer equipped with a QCI-CryoProbe. The spectrum was acquired with 112 scans and 256 increments in the F1 dimension. The spectral width was 7716 Hz in the ^{1}H dimension and 42277 Hz in the ^{13}C dimension. The acquired data matrix was 2048 × 256 points and the spectrum was processed using a final matrix size of 2K × 1K.
	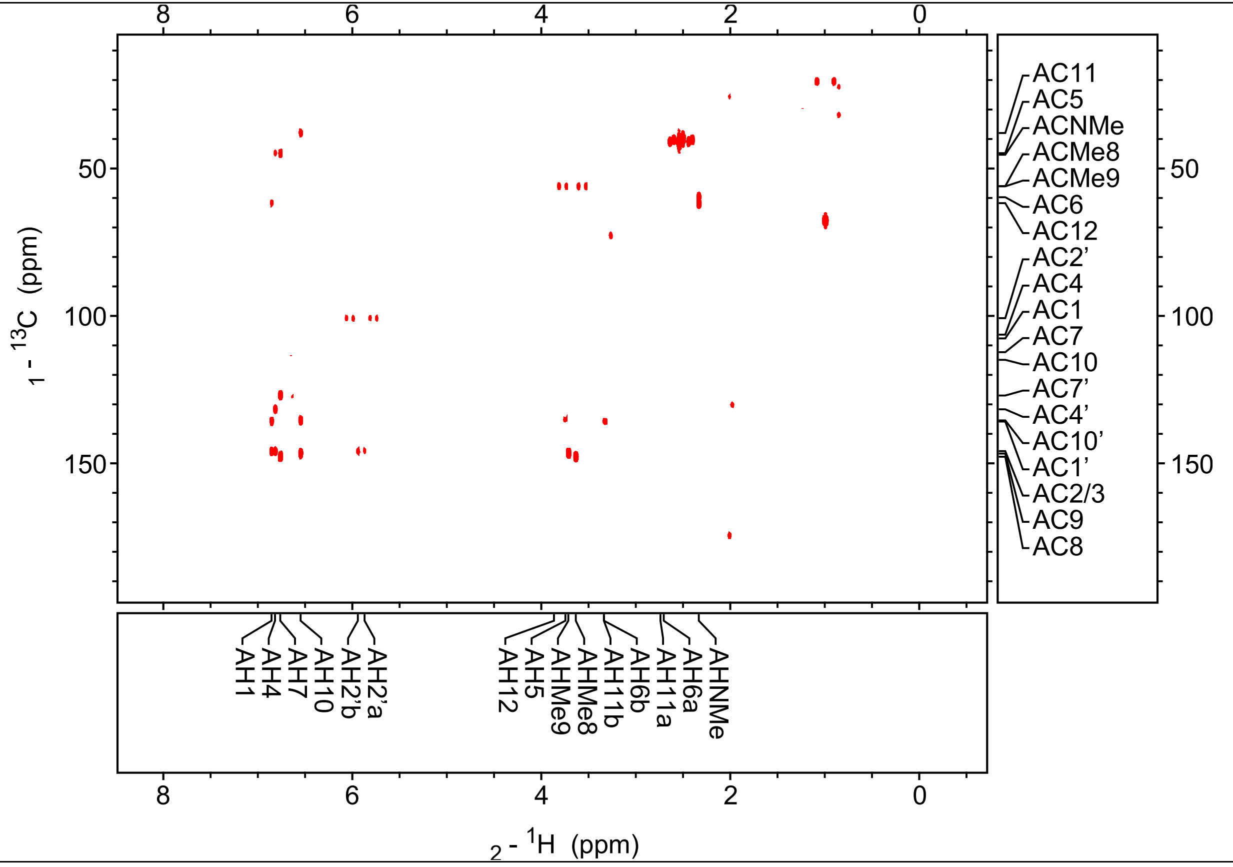
